# Identification of Homogeneous Subgroups from Resting-State fMRI Data

**DOI:** 10.3390/s23063264

**Published:** 2023-03-20

**Authors:** Hanlu Yang, Trung Vu, Qunfang Long, Vince Calhoun, Tülay Adali

**Affiliations:** 1 Department of Computer Science and Electrical Engineering, University of Maryland Baltimore County, Baltimore, MD 21250, USA; 2 Tri-Institutional Center for Translational Research in Neuroimaging and Data Science (TReNDS), Georgia State University, Georgia Institute of Technology, and Emory University, Atlanta, GA 30303, USA

**Keywords:** precision medicine, subgroup identification, ICA, constrained ICA, resting-state fMRI

## Abstract

The identification of homogeneous subgroups of patients with psychiatric disorders can play an important role in achieving personalized medicine and is essential to provide insights for understanding neuropsychological mechanisms of various mental disorders. The functional connectivity profiles obtained from functional magnetic resonance imaging (fMRI) data have been shown to be unique to each individual, similar to fingerprints; however, their use in characterizing psychiatric disorders in a clinically useful way is still being studied. In this work, we propose a framework that makes use of functional activity maps for subgroup identification using the Gershgorin disc theorem. The proposed pipeline is designed to analyze a large-scale multi-subject fMRI dataset with a fully data-driven method, a new constrained independent component analysis algorithm based on entropy bound minimization (c-EBM), followed by an eigenspectrum analysis approach. A set of resting-state network (RSN) templates is generated from an independent dataset and used as constraints for c-EBM. The constraints present a foundation for subgroup identification by establishing a connection across the subjects and aligning subject-wise separate ICA analyses. The proposed pipeline was applied to a dataset comprising 464 psychiatric patients and discovered meaningful subgroups. Subjects within the identified subgroups share similar activation patterns in certain brain areas. The identified subgroups show significant group differences in multiple meaningful brain areas including dorsolateral prefrontal cortex and anterior cingulate cortex. Three sets of cognitive test scores were used to verify the identified subgroups, and most of them showed significant differences across subgroups, which provides further confirmation of the identified subgroups. In summary, this work represents an important step forward in using neuroimaging data to characterize mental disorders.

## 1. Introduction

With the increasing availability of large-scale multi-subject data, precision medicine is set to change modern medical care [1]. Traditional symptom-based medical care, in which clinical decisions are made based on the data collected from a large population of patients, is envisioned to be replaced by a more individual-based approach where personalized treatment is grounded in deep assimilation of a subgroup of patients carrying unique disease characteristics [2]. By taking into consideration the key characteristics shared by specific subgroups of patients, precision medicine aims to target treatments that benefit the patients the most and with sparse side effects. The core challenge of precision medicine is to classify patients into homogeneous subgroups based on the biomarkers of a certain disease, where individuals share homogeneity within a subgroup and express heterogeneity across subgroups. This becomes more challenging for patients with psychiatric disorders because the etiology of a majority of neuropsychiatric illnesses is unclear [3,4,5,6], even though studies show that numerous subtypes exist within broad neuropsychiatric disorder domains including schizophrenia [7], bipolar disorder [8], major depression [9,10], and autism [11]. Traditionally, parsing patients with mental disorders is based on primarily subjective measures including descriptive psychopathology, which originates from the expression of each individual, behavioral characteristics, cognitive test scores, and other related indices [12,13]. The classification accuracy is limited due to the limitation of identifying internal abnormalities based on external symptoms and the uncertain relationship between subtypes and post hoc descriptions [14].

Functional magnetic resonance imaging (fMRI), reflecting neural activity changes in an area of the brain by measuring the blood-oxygenation-level-dependent (BOLD) signal, has been attractive for understanding neurological changes associated with a broad range of psychiatric disorders [15]. Besides the insights provided by fMRI, research also shows that the functional connectivity profiles captured by fMRI preserve subject variability and act as fingerprints [16]. The sustainability and reproducibility of the identified functional profiles from fMRI provide a valuable opportunity for classifying psychiatric patients into subgroups by analyzing their brain functions.

Independent component analysis (ICA) has been successfully applied to fMRI analysis for estimating functional networks in a data-driven manner [17,18,19]. Hence, ICA can identify putative biomarkers of multiple neuropsychiatric disorders effectively [19,20,21,22,23,24,25]. ICA decomposes a subject’s brain activities into a series of functional networks that are maximally independent. Analyzing individual-level ICA results from a large-scale multi-subject dataset can be complicated because the components across subjects are not aligned due to the intrinsic sign and permutation ambiguity of ICA, which makes it difficult to draw group inferences [25]. Group ICA (GICA) [26] generalizes ICA to provide group inferences for multi-subject analysis. GICA performs a first-level PCA on individual subject data followed by a second-level PCA on the vertically concatenated data. A single ICA estimation is performed only on the aggregated group-level data. The subject-specific result is achieved by the back-reconstruction step in GICA, which allows for inter-subject comparison of the spatial and temporal results. However, the assumption of common group-level spatial maps makes GICA less competitive than another extension of ICA, independent vector analysis (IVA), in preserving inter-subject variability [27,28,29,30,31]. IVA [32] extends ICA to multi-subject analysis by effectively making use of the dependence across datasets [25,33]. By exploiting the multivariate information across the subjects, IVA allows the subject datasets to fully interact with each other [25]; this makes IVA a good candidate for subgroup identification. Studies on the application of IVA to subgroup identification yield promising results that meaningful functional networks are detected and show significant group differences across the identified subgroups [34,35]. However, IVA is computationally expensive, and its performance degrades as the number of datasets increases [34]. In [34,35], both of the studies implemented IVA on only 50 subjects due to the computational complexity, which limits the potential of identifying subgroups from a large-scale (a couple of hundred to thousand of subjects) dataset.

To balance the trade-off between the accuracy and complexity of an algorithm, adding a constraint is a general solution when certain domain knowledge is accessible. Compared with general ICA, constrained ICA (c-ICA) allows multi-subject datasets to be connected through the constraints and the components to be aligned across subject-level ICA analyses, which provides a foundation for identifying subgroups from a large-scale dataset. Studies on c-ICA show that providing robust prior information increases c-ICA overall performance in source separation [36,37,38,39,40,41]. Compared with IVA, c-ICA overcomes the computational complexity caused by over-parameterization due to its flexibility as it can be applied to individual subjects. Compared with GICA, c-ICA better preserves inter-subject variability as it analyzes individual subjects separately. Therefore, c-ICA is a good candidate for subgroup identification because it is able to leverage the rich information of subgroups from a large-scale dataset while maintaining a low computational cost.

This study aims to identify subgroups from a large-scale multi-subject resting-state fMRI dataset consisting of 464 subjects, including patients with schizophrenia (176), psychotic bipolar disorder (159), and schizoaffective disorder (129). The identification of subgroups from fMRI or other large datasets is a crucial problem that has not yet been sufficiently addressed. To properly address this issue, we propose a pipeline that makes use of functional activity maps for subgroup identification with the Gershgorin disc theorem, which we call fSIG. The proposed pipeline uses a c-ICA- and Gershgorin disc-based method to identify homogeneous subgroups within which subjects have similar functional network activity patterns. Additionally, we present a flexible constrained algorithm, c-EBM, using the entropy bound minimization (EBM) technique [42]. There are limitations of current c-ICA algorithms that impose an orthogonality constraint on their demixing matrices and only use fixed nonlinearity, which limits the solution space and also performance [36,37,38,39,40,41]. c-EBM overcomes the aforementioned limitations by providing a flexible density matching and decoupling for the demixing matrix without the orthogonality constraint. To extract reliable resting-state networks as references for c-EBM, group ICA-EBM [26,42] is incorporated into the fSIG pipeline. We demonstrate that results from c-EBM capture inter-subject variability and can be used for subgroup identification studies. Preliminary work using the Gershgorin disc theorem for subgroup identification study is presented in our previous work [35], which is incorporated within the fSIG pipeline along with c-EBM in this work. Our results show that the fSIG pipeline is able to identify homogeneous subgroups that show significant activity differences in multiple meaningful brain areas such as the dorsolateral prefrontal cortex, anterior cingulate cortex, superior temporal gyrus, etc. These areas have been connected with functional deficits in psychiatric disorders in multiple prior studies [43,44,45]. Compared with subgroups identified by the method in [34], results from fSIG show various advantages including: (1) better subgroup structures such that subjects within each subgroup have higher correlations than the subjects outside the subgroup in their functional network activities; (2) more meaningful brain areas that show significant group differences. Besides using neuroimaging data, three sets of cognitive tests scores: Social Functioning Scale (SFS) [46], Brief Assessment of Cognition in Schizophrenia (BACS) [47], and Positive and Negative Syndrome Scale (PANSS) [48] are statistically analyzed in order to validate the identified subgroups from different perspectives. Results from fSIG show significant group differences on most cognitive test scores across the subgroups identified with neuroimaging data, which increases our confidence that the identified subgroups are meaningful.

The rest of this paper is organized as follows: methods used to identify subgroups, including ICA-EBM, c-EBM, and the Gershgorin disc-based method for subgroup identification are presented separately in Section 2.1, Section 2.2 and Section 2.3. The data used in this study and the preprocessing are introduced in Section 2.5. The template generation steps are listed in Section 2.6. The implementation of c-EBM on subgroup identification and its corresponding results are presented in Section 3, followed by discussions in Section 4.

## 2. Materials and Methods

### 2.1. ICA-EBM

The basic noiseless ICA problem can be modeled as [25]

(1) x(v)=As(v),1≤v≤V,

where $x\left(v\right)={\left[x_{1}\left(v\right),\cdots ,x_{N}\left(v\right)\right]}^{⊤}$ is an observation vector (e.g., from a single subject) at sample index *v* (superscript ⊤ represents transpose); $s\left(v\right)={\left[s_{1}\left(v\right),\cdots ,s_{N}\left(v\right)\right]}^{⊤}$ are *N* statistically independent, zero mean, and unit variance latent sources; and $A\in R^{N\times N}$ is an unknown invertible mixing matrix. Given the model, ICA estimates the latent sources by finding a demixing matrix $W\in R^{N\times N}$ such that the estimates $y\left(v\right)={\left[y_{1}\left(v\right),\ldots ,y_{N}\left(v\right)\right]}^{⊤}$, where y(v)=Wx(v), are maximally independent. To simplify the notation, the sample index *v* is suppressed in the rest of this paper. The ICA cost function, J(W), can be represented as the mutual information among *N* source estimates $y_{n}$ for n=1,⋯,N, which can be written as a function of the demixing matrix W [25]

(2) $$\begin{array}{cc} J(W) & =\sum_{n=1}^{N}H\left(y_{n}\right)-\log\left|\det\left(W\right)\right|-H\left(x\right), \end{array}$$

where $H(y_{n})$ is the (differential) entropy of the nth estimation $y_{n}$, log|det(W)| is a regularization term preventing the demixing matrix W having small determinant and the cost function being minimized by only scaling the estimates. The last term, H(x), is the entropy of x and is constant with respect to W.

Sources can be estimated by minimizing J(W) with respect to W. In order to calculate the entropy of the nth estimate, $H\left(y_{n}\right)=-E\left[\log p_{y_{n}}\left(y_{n}\right)\right]$, the probability density function $p_{y_{n}}\left(y_{n}\right)$ is required. However, the estimation of a probability density function can be computationally complicated [49]. Therefore, finding a flexible way to estimate the entropy approximation is important for ICA. ICA by entropy bound minimization (EBM) estimates entropy by utilizing a finite number of predefined measuring functions coming from various distributions and the maximum entropy principle. Previous research [50] shows that, with fMRI data, EBM provides better estimations of functionally relevant components than other ICA algorithms such as Infomax.

In order to obtain a better model match for each source and simplify the optimization procedure, a decoupling step is performed to divide the matrix optimization problem into a series of vector optimization problems. One way of applying decoupling is to constrain the demixing matrix W to be orthogonal [49]. However, the regularization term becomes fixed, and the solution space shrinks with the orthogonality constraint. Another way of implementing decoupling procedure on (2) is through the method proposed in [51], which does not constrain W to be orthogonal. The original problem is simplified to minimize the mutual information among the estimated sources with respect to each row vector $w_{n}$, based on which (2) can be rewritten as

(3) $$\begin{array}{cc} J_{n}\left(w_{n}\right) & =H\left(y_{n}\right)-\log\left|d_{n}^{⊤}w_{n}\right|+C, \end{array}$$

where $d_{n}^{⊤}$ is a unit vector that is perpendicular to all the rows of the demixing matrix W except $w_{n}$[51] and *C* is a constant that does not depend on $w_{n}$. With the decoupling procedure, the ICA-EBM cost function can be written as

(4) $$\begin{array}{cc} J_{n}\left(w_{n}\right) & =-O_{m(n)}\left\{E\left[G_{m(n)}\left(y_{n}\right)\right]\right\}-\log\left|d_{n}^{⊤}w_{n}\right|+C_{1}, \end{array}$$

where $O_{m(n)}\left\{E\left[G_{m(n)}\left(y_{n}\right)\right]\right\}$ measures the negentropy of the nth estimation and can be solved numerically as in [42]; $G_{m(n)}$, for m=1,⋯,M, is a selected measure function from a set of *M* (M=4 in this paper) measuring functions G(x): $x^{4}$, $\frac{\left|x\right|}{1+|x|}$, $\frac{x|x|}{10+|x|}$, $\frac{x}{1+x^{2}}$; the value of O(·) is calculated beforehand to alleviate the followed calculation; and $C_{1}$ is a constant with respect to $w_{n}$.

### 2.2. Constrained EBM

ICA has been widely used as a fully data-driven method for fMRI analysis due to its minimal assumptions about the data. When reliable references are available, the source separation performance of ICA can be improved by leveraging the prior information through incorporating references such as spatial activation maps into the ICA cost function [36,37,38,39,40,41].

For a given observed dataset comprising *V* samples, the estimations are given by the matrix equation Y=WX, with $Y,X\in R^{N\times V}$. The reference of the nth source estimate $y_{n}={\left[y_{n}\left(1\right),\ldots ,y_{n}\left(V\right)\right]}^{⊤}\in R^{V}$ is denoted by $r_{n}\in R^{V}$. The constraint function $h_{n}\left(\cdot \right)$ is defined as

(5) $$h_{n}\left(r_{n},y_{n}\right)=\theta_{n}-\epsilon \left(r_{n},y_{n}\right)\leq 0,$$

where $\epsilon (r_{n},y_{n})$ quantifies the similarity between the nth estimates and its corresponding reference and $\theta_{n}$ is the constraint parameter that tolerates the level of deviation of $y_{n}$ from $r_{n}$. The absolute value of the Pearson correlation is used as the measurement of similarity:

(6) $$\epsilon \left(r_{n},y_{n}\right)=\left|\mathrm{corr}\left(r_{n},y_{n}\right)\right|=|\frac{r_{n}^{⊤}y_{n}}{∥r_{n}∥\;∥y_{n}∥}|\in \left[0,1\right].$$

With the decoupling procedure in (4), c-EBM is able to apply constraints to individual sources without assuming the demixing matrix W to be orthogonal. The value of $\theta_{n}$ is restricted between 0 and 1 and can be determined based on specific applications. Higher values of $\theta_{n}$ provide estimates that are highly similar to the references, which can be helpful when keeping the variability of the datasets is not the priority. On the other hand, estimates from c-EBM with lower values of $\theta_{n}$ are less similar to the references but better at preserving the variability across datasets, which is preferred for subgroup identification.

We apply an augmented Lagrangian-based approach to incorporate the inequality constraint (5) into the cost function (4). A slack variable *z* is defined as $h_{n}\left(r_{n},y_{n}\right)+z^{2}=0$ to replace the inequality constraint with an equality constraint. The cost function of c-EBM can be written as [40]

(7) $$\begin{array}{cc} J_{n}^{c}\left(w_{n},\mu_{n}\right) & =J_{n}\left(w_{n}\right)+\frac{1}{2\gamma }\left[\max{\left\{0,\left[\gamma h_{n}\left(r_{n},y_{n}\right)+\mu_{n}\right]\right\}}^{2}-\mu_{n}^{2}\right], \end{array}$$

where $\mu_{n}$ is a Lagrangian multiplier and $\gamma \in R^{+}$ is a learning parameter. Gradient descent approach is used for minimizing $J_{n}^{c}$ with respect to $w_{n}$, with the update

(8) $$\begin{array}{cc} ▵w_{n} & =w_{n}^{i+1}-w_{n}^{i}\\ \; & \propto \frac{\partial J_{n}^{c}\left(w_{n}\right)}{\partial w_{n}}=-O_{m(n)}^{\prime }\left\{E\left[G_{m(n)}\left(y_{n}\right)\right]\right\}E\left[g_{m(n)}\left(y_{n}\right)X\right]\\ \; & \;\;\;\;-\frac{d_{n}}{d_{n}^{⊤}w_{n}}-\max(0,[\gamma_{n}h_{n}\left(r_{n},y_{n}\right)+\mu_{n}])\mathrm{sign}\left(r_{n}^{⊤}y_{n}\right)\left(\frac{1}{V}Xr_{n}\right), \end{array}$$

where *i* denotes the iteration index, and $O{\left(\cdot \right)}^{\prime }$ and $g_{m(n)}$ are the first order derivatives of O(·) and $G_{m(n)(\cdot )}$, respectively. In each iteration, the Lagrange multiplier $\mu_{n}$ is updated by

(9) $$\mu_{n}\leftarrow \max\left\{0,\left[\gamma_{n}h_{n}\left(r_{n},y_{n}\right)+\mu_{n}\right]\right\}.$$

### 2.3. Subgroup Identification

Functional connectivity profiles estimated using fMRI data are shown to be robust and stable for identifying subject variability, similar to fingerprints. From this point of view, a homogeneous subgroup (SG) can be defined as group subjects that have similar functional network activity patterns, i.e., higher correlation within the group than the subjects outside the group. Each component from the ICA results represents an activation map of a functional network for a given subject. By taking advantage of the prior information provided by the references $r_{n}$, c-EBM is able to align the components across subjects which alleviates the post-process alignment for subgroup study. Group ICA [26] is used to extract reliable resting-state networks as reference followed by performing subject-level analysis with c-EBM.

Subject-wise c-EBM is applied to *K* subjects separately. Because each subject is a dataset for c-EBM, in this paper, we use *K* subjects and *K* dataset interchangeably. To evaluate the similarity of a specified functional network across *K* subjects, let us recall from (1) the ICA model for the kth subject

(10) $$x^{\left[k\right]}\left(v\right)=A^{\left[k\right]}s^{\left[k\right]}\left(v\right),\;1\leq v\leq V.$$

The vth sample of the source vector, $s^{\left[k\right]}\left(v\right)={\left[s_{1}^{\left[k\right]}\left(v\right),\ldots ,s_{N}^{\left[k\right]}\left(v\right)\right]}^{⊤}$, is a realization of the random vector $s^{\left[k\right]}\in R^{N}$. We define the nth source component vector (SCV) as $s_{n}={\left[s_{n}^{\left[1\right]},\cdots ,s_{n}^{\left[K\right]}\right]}^{⊤}$, which is a random vector independent of all other SCVs. Accordingly, the estimate of the nth SCV is denoted by $y_{n}={\left[y_{n}^{\left[1\right]},\cdots ,y_{n}^{\left[K\right]}\right]}^{⊤}$. For the vth sample, the corresponding realization of the nth estimated SCV is $y_{n}\left(v\right)={\left[y_{n}^{\left[1\right]}\left(v\right),\cdots ,y_{n}^{\left[K\right]}\left(v\right)\right]}^{⊤}$, where $y_{n}^{\left[k\right]}\left(v\right)=w_{n}^{{\left[k\right]}^{⊤}}x^{\left[k\right]}\left(v\right)$. Thus, for a total of *V* samples, the estimated sample covariance matrix of the nth SCV is given by

$$\begin{array}{c} {\hat{C}}_{n}=\frac{1}{V-1}Y_{n}Y_{n}^{⊤},\;\mathrm{where}\;Y_{n}=\left[\begin{array}{cccc} y_{n}^{\left[1\right]}\left(1\right) & y_{n}^{\left[1\right]}\left(2\right) & \ldots & y_{n}^{\left[1\right]}\left(V\right)\\ y_{n}^{\left[2\right]}\left(1\right) & y_{n}^{\left[2\right]}\left(2\right) & \ldots & y_{n}^{\left[2\right]}\left(V\right)\\ \vdots & \vdots & \ddots & \vdots \\ y_{n}^{\left[K\right]}\left(1\right) & y_{n}^{\left[K\right]}\left(2\right) & \ldots & y_{n}^{\left[K\right]}\left(V\right) \end{array}\right]\in R^{K\times V}. \end{array}$$

We note that ${\hat{C}}_{n}$ provides the correlation information among *K* subjects for a given functional network. Subjects that have similar functional network activation patterns will show higher correlation value within themselves, which forms block structures in ${\hat{C}}_{n}$.

Subgroups are defined based on the correlation values of subjects with similar functional network activation patterns. Specifically, these subgroups are identified as the diagonal blocks within the sample covariance matrix ${\hat{C}}_{n}$, where the entries within each block represent the correlations between subjects in that subgroup. To obtain a sample covariance matrix with diagonal block structures, we permute ${\hat{C}}_{n}$ based on the subjects’ indices from the subgroup identification results. Therefore, we can define the sample covariance matrix of the nth SCV which has *B* diagonal blocks as ${\hat{C}}_{n}=\mathrm{blkdiag}\left(G_{n}^{1},\ldots ,G_{n}^{B}\right)$, where $G_{n}^{b}\in R^{g_{b}\times g_{b}}$, for b=1,…,B, represents the correlation coefficients between the $g_{b}$ subjects within one subgroup. In situations where there exists a distinct group of subjects that do not display correlations with any other subjects or belong to any subgroups, the final diagonal block in ${\hat{C}}_{n}$ may consist of an identity matrix. Typically, the diagonal-block structure has off-diagonal block entries with lower values than those within the diagonal block. This structure indicates that the subjects within the corresponding subgroup exhibit a high correlation with one another.

The subgroup identification process consists of two steps: (I) cluster SCVs based on ${\hat{C}}_{n}$ such that SCVs with similar activation patterns across subjects will be in the same cluster Φ; (II) identify homogeneous subgroups based on the aggregated covariance matrix $\bar{C}$ calculated from the ith cluster of the SCVs, $\Phi_{i},\;1\leq i\leq I$, where *I* is the total number of clusters and $\bar{C}=1/M_{i}\sum_{m\in \Phi_{i}}{\hat{C}}_{m}$ is the mean matrix of the sample covariance matrices in clusters $\Phi_{i}$ and $M_{i}$ is the total number of SCVs included in $\Phi_{i}$.

**Figure 1:**
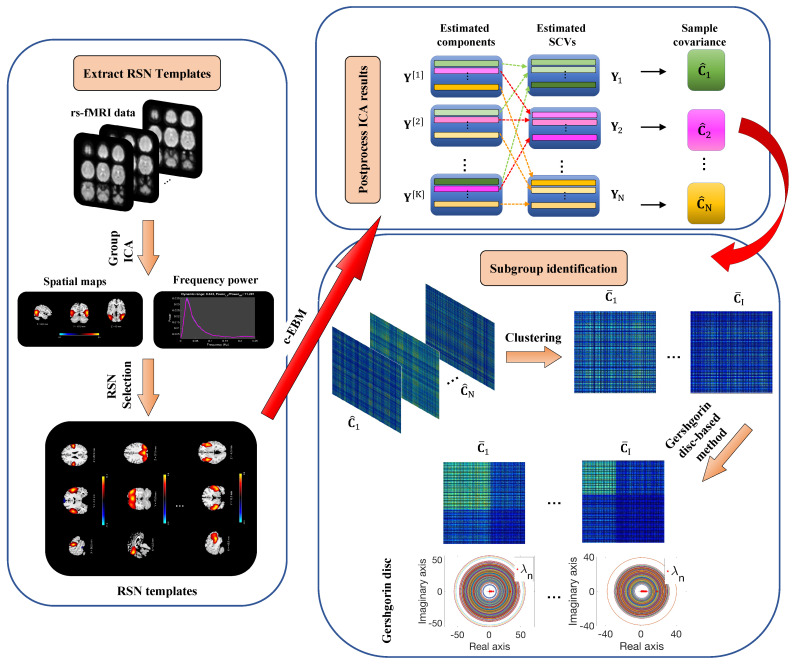
Flowchart of the proposed subgroup identification pipeline, fSIG. **Step 1:** Extract the resting-state network (RSN) templates by applying group ICA-EBM on an independent dataset (COBRE). **Step 2:** Apply c-EBM on subjects from BSNIP dataset with the extracted RSN templates and form the sample covariance matrix ${\hat{C}}_{n}$ from the estimated SCV $Y_{n}$. **Step 3:** Cluster the sample covariance matrix ${\hat{C}}_{n}$ based on their similarity of connectivity patterns in $Y_{n}$ and calculate the aggregated sample covariance matrix ${\bar{C}}_{i}$ by taking the average of ${\hat{C}}_{n}$ within the same cluster $\Phi_{i}$. The Gershgorin disc-based method is applied to ${\bar{C}}_{i}$ to identify the number of potential homogeneous subgroups and the subjects that belong to each subgroup. In the plots of Gershgorin discs, the red dots and the circles are the actual eigenvalues $\lambda_{n}$ and the Gershgorin discs of ${\bar{C}}_{i}$.

Because a homogeneous subgroup represents a group of subjects that have a similar activation pattern in one or more functional networks, Step I uses the *k*-means algorithm to separate SCVs into different clusters. The correlations of subjects’ activation patterns for the SCVs within a cluster are optimized to the centroid of the cluster. The optimal number of centroids is estimated to maximize the modularity of the aggregated sample covariance matrix $\bar{C}$. Process in Step I is inspired by the exemplars concept from [52], where a similar process was applied to cluster dynamic functional network connectivity (dFNC) windows. As both the covariance matrix of SCVs and dFNC windows reflect subjects’ connectivity patterns, the exemplars concept is a good candidate for clustering SCVs. Step II reveals subgroup structures from $\bar{C}$ by implementing Gershgorin disc-based method [35], where the number of eigenvalues of $\bar{C}$ located outside the smallest Gershgorin disc determines the number of subgroups and the corresponding eigenvectors identify the index of the subjects that belong to each subgroup. The identified subgroups have higher intragroup similarity than the rest of the subjects from the perspective of their functional networks’ spatial activation patterns. The flowchart of the proposed subgroup identification pipeline, fSIG, is displayed in Figure 1.

### 2.4. Subgroup Validation

The validation of identified subgroups is based on statistical tests of spatial maps and three sets of cognitive scores. To compare the spatial activation patterns of the RSNs that show group differences between identified subgroups, a two-sample *t*-test is performed on the activation value of each voxel across subjects belonging to each subgroup, and results are plotted on the corresponding *t*-maps. False discovery rate (FDR) correction [53] is implemented on all *t*-test results. Because there exists a possibility that more than one brain area that shows group differences between the identified subgroups, global difference map (GDM) [54] is used to summarize the distinctive power of each cluster. GDM is defined as

(11) $$\begin{array}{c} T_{\mathrm{GDM}}^{\Phi_{i}}=\sum_{q=1}^{Q_{i}}\frac{|t_{q}|}{\sum_{l=1}^{Q_{i}}t_{l}}T_{q}^{\Phi_{i}}, \end{array}$$

where $t_{q}$ and $T_{q}^{\Phi_{i}}$ are the *t*-statistic and the *t*-map of qth SCV that shows significant group difference between identified subgroups in the ith cluster $\Phi_{i}$ separately.

### 2.5. Data Preprocessing

#### 2.5.1. Resting-State fMRI

The resting-state fMRI datasets and the corresponding behavioral variables are from the Bipolar-Schizophrenia Network on Intermediate Phenotypes (B-SNIP) [55,56]. Identical diagnostic and recruitment approaches were applied to all recruited subjects at multiple sites (Baltimore, Chicago, Dallas, Detroit, and Hartford). All subjects at each site underwent a single 5 min run of resting-state fMRI on a 3-T scanner. Subjects were instructed to keep their eyes open, focus on a crosshair displayed on a monitor, and remain still during the entire scan [55].

We removed the first three time points and performed head motion correction followed by the slice-timing correction. The corrected fMRI data were then warped into the standard Montreal Neurological Institute (MNI) space through an echo-planar imaging template and then were resampled to 3 × 3 × 3 mm${}^{3}$ isotropic voxels. The resampled fMRI data were further smoothed using a Gaussian kernel with a full width at half maximum (FWHM) equal to 6 mm. Quality control [57] was applied to select subjects. A total of 1143 subjects passed quality control, comprising healthy controls (229 subjects), patients with various diagnoses (464 subjects), and relatives of the patients (450 subjects). In this study, only data from 464 patients are used, which included 176 individuals with schizophrenia, 159 with psychotic bipolar disorder, and 129 with schizoaffective disorder.

#### 2.5.2. Cognitive Test Scores

Three sets of cognitive test scores, the Social Functioning Scale (SFS) [46], the Brief Assessment of Cognition in Schizophrenia (BACS) [47], and the Positive and Negative Syndrome Scale (PANSS) [48], were collected from the same subjects. The SFS score evaluates the key social skills and performance of individuals. Studies of functional imaging have shown that there is a significant connection between social functioning and functional brain imaging [58,59,60,61]. The BACS score includes six subtests and an overall composite score that quantify the neurocognitive deficits of schizophrenia patients. The six subtests, list learning, digit sequencing, token motor, verbal fluency, symbol coding, and the tower of London, separately assess the functional abilities of verbal memory, working memory, motor speed, verbal fluency, attention, and speed of information processing, and executive function [47]. The quantification of functional abilities has provided insightful guidance on determining the correlation between the altered functional connectivity strength and the cognitive function decrease in individuals with psychosis [62,63,64]. The PANSS score includes 30 disparate symptoms observed in psychotic patients and is evaluated with a scale ranging from 1 to 7. The scores are able to consistently reflect three dimensions of the symptom: positive, negative, and general [65]. PANSS has been used to quantify the differences of functional networks across multiple psychotic probands [66,67]. The aforementioned three sets of cognitive tests give a thorough validation and assessment of the identified subgroups. For any missing cognitive test data of the subjects, they are padded with the mean of that specific test or subtest from all the subjects with the available test data.

### 2.6. Reference Generation

#### 2.6.1. Data Acquisition and Preprocessing

The reference signals are generated from a large-scale resting-state fMRI data including 91 healthy controls (average age: 38±12) collected by Center of Biomedical Research Excellence (COBRE) [68,69,70], which is accessible from (https://coins.trendscenter.org/, accessed on 10 January 2023). During the scan, participants followed the instructions to keep their eyes open and stare passively at a central fixation cross. All resting-state fMRI data were collected on a single 3-Tesla Siemens Trio scanner with a 12-channel radio frequency coil, TE = 29 ms, TR = 2 s, flip angle =$75^{\circ }$, slice gap = 1.05 mm, slice thickness = 3.5 mm, voxel size =3.75×3.75×4.55 mm${}^{3}$. The fMRI scans were obtained over five minutes with a sampling period of 2 seconds, which generates 150 time points for each subject. The first six time points were removed to address the T1-effect. We performed motion correction, slice time correction, and spatial normalization and re-sampled each subject’s data to 3×3×3 mm${}^{3}$ yielding 53×63×46 voxels in total.

#### 2.6.2. Model Order and RSNs Selection

Group ICA using EBM algorithm [26,42] is applied to the 91 subjects with model order set as 100. Because model order is an important step of the model match for ICA, we designed a thorough process for order selection based on the concept of cross-ISI [71]. Cross-ISI, which is denoted as ${\mathrm{ISI}}_{ij}^{C}=\mathrm{ISI}\left(G^{ij}\right)$, measures the consistency of the ith run to the jth run, where

(12) $$\begin{array}{cc} \mathrm{ISI}\left(G^{ij}\right)=\frac{1}{2N(N-1)} & (\sum_{n=1}^{N}\left(\frac{\sum_{m=1}^{N}\left|g_{nm}\right|}{\max_{p}\left|g_{np}\right|}-1\right)+\left(\sum_{m=1}^{N}\left(\frac{\sum_{n=1}^{N}\left|g_{nm}\right|}{\max_{p}\left|g_{mp}\right|}-1\right)\right), \end{array}$$

with $G^{ij}=A_{i}W_{j}$ with elements $g_{nm}$[71,72], where $A_{i}=W_{i}^{-1}$ is the inverse matrix of the demixing matrix of the ith run, |·| is the absolute value, and $W_{j}$ is the estimated demixing matrix of jth(j≠i) run. The cross-ISI of the ith run, which measures the consistency of the ith run to the other runs (*R* runs in total), is calculated by averaging all its pairwise cross-ISI, which is defined as

(13) $$\begin{array}{c} {\mathrm{ISI}}_{i}^{C}=\frac{1}{R-1}\sum_{j=1,\;j\neq i}^{R}{\mathrm{ISI}}_{ij}^{C}. \end{array}$$

**Figure 2:**
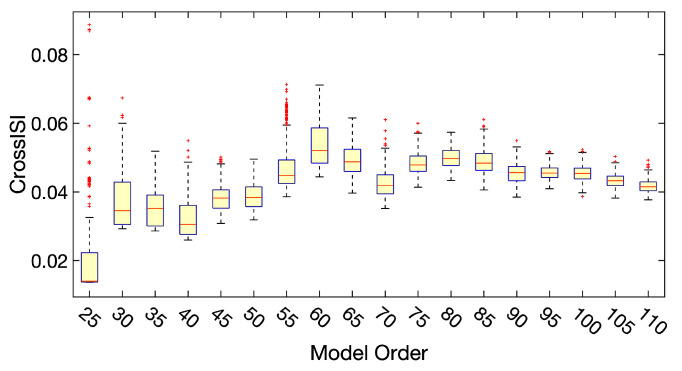
Using Cross-ISI as an objective index of choosing an appropriate model order of group ICA-EBM. The estimated model orders ranging from 25 to 110 with step size 5 and 300 runs of ICA-EBM with random initialization are applied to each one of the estimated model orders. The final model order is decided by considering various factors, including the lowest Cross-ISI value, the variance of the Cross-ISI, and the anatomical and functional properties of each model order result. For each box, the lines from top to bottom represent the maxim, the 25th and 75th percentiles, the median, and the minimum of the Cross-ISI, respectively. The red plus signs represent outliers.

The final model order is decided by the following steps:

1. Estimate the model order by entropy-rate based order selection using the finite memory length model (ER-FM) and autoregressive model (ER-AR) [73], as 71.73±7.76 and 77.29±7.74, respectively;
2. Test the estimated model order ranging from 25 to 110 with step size 5;
3. Perform 300 runs of ICA-EBM with random initialization on each one of the estimated model orders;
4. Calculate Cross-ISI for all the runs. The distribution of Cross-ISI for all the orders is displayed in Figure 2;
5. Select model orders that have relatively small values and small variance of Cross-ISI;
6. Select the best run that has the smallest Cross-ISI from the selected model orders;
7. Inspect the results based on the visualization of spatial activation of functional networks and the corresponding spectral summary.

**Figure 3:**
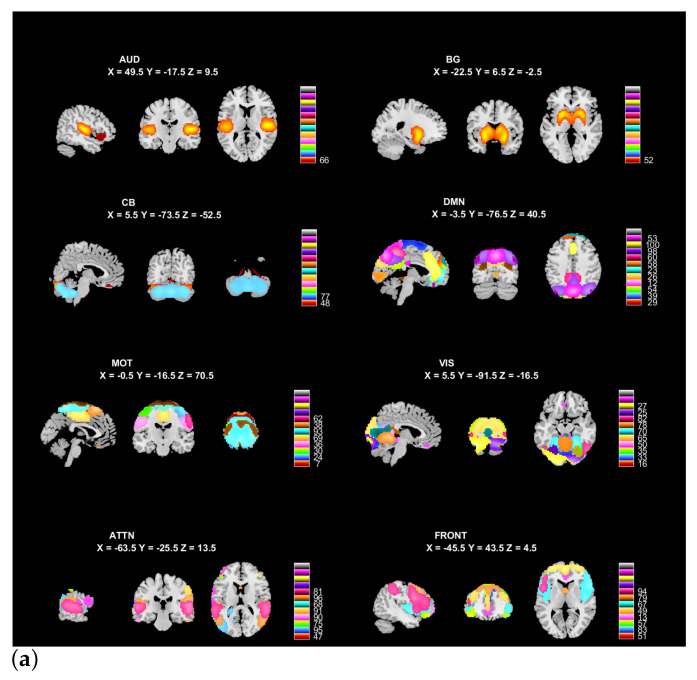

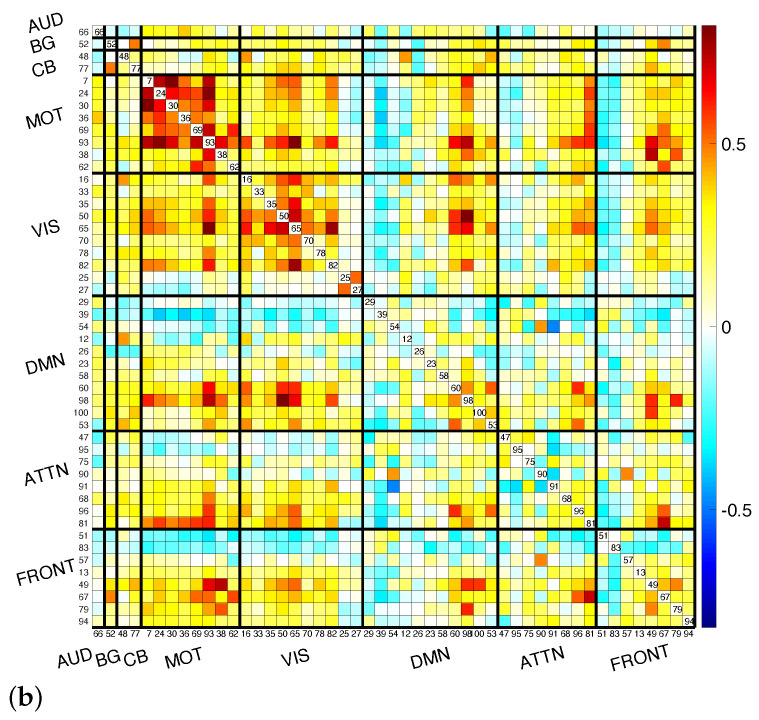
Selected functional network references and the corresponding aggregate functional network connectivity matrix. (**a**) Visualization of the selected functional network references, which were separated into eight functional areas based on their anatomical and functional properties. In each functional area, one color in the composite maps corresponds to one component. (**b**) Aggregated functional network connectivity matrix. Pairwise correlations between resting-state networks’ time courses are first Fisher z-transformed and averaged across all subjects, then inverse z-transformed back to display.

Given the fact that GICA assumes a common spatial space for all the subjects, we choose a higher model order of 100 to better preserve subject variability. The final 49 resting-state networks (RSNs) components used as the functional network templates are selected based on the inspection of their spatial maps, power ratio between low-frequency and high-frequency signals, and location of brain regions. Those components are organized into eight functional areas with respect to their functional and anatomical properties [74,75], namely, auditory (AUD: 1 RSN), sensorimotor (MOT: 8 RSNs), visual (VIS: 10 RSNs), default-mode (DMN: 11 RSNs), attentional (ATTN: 8 RSNs), and frontal (FRONT: 8 RSNs), cerebellar (CB: 2 RSNs), and basal ganglia (BG: 1 RSN) networks. The visualization of the selected RSNs and their aggregated functional network connectivity (FNC) matrix are given in Figure 3a and Figure 3b, respectively.

## 3. Results

With the aforementioned RSN templates, c-EBM is applied to all patients (464 subjects) separately with the constraint parameter set as 0.3. The relatively lower constraint value is used to preserve the inter-subject variability with respect to the active patterns of the spatial maps.

**Figure 4:**
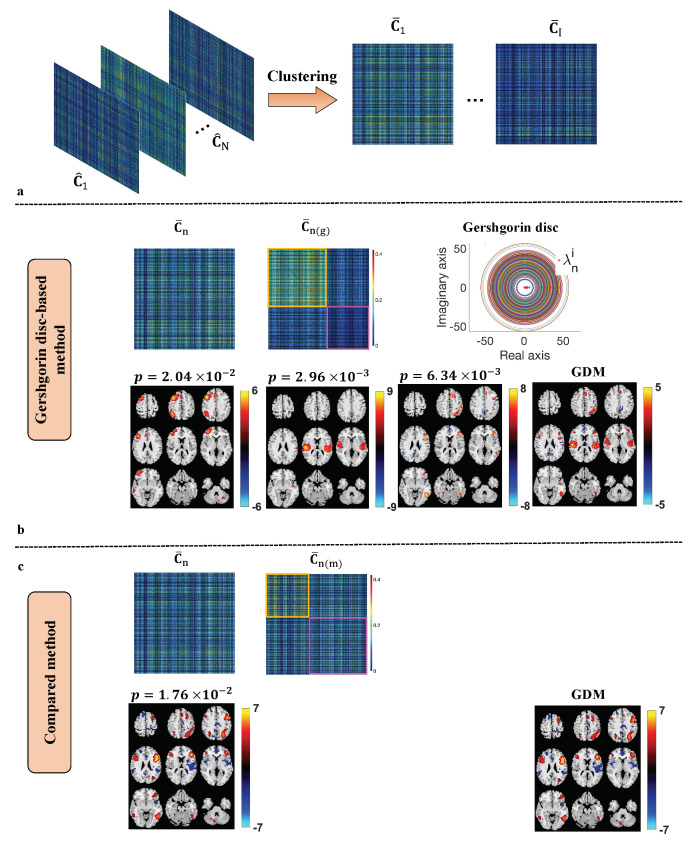
Performance comparison between the Gershgorin disc-based method and the compared method in [34] on the aggregated matrix $\bar{C}$. The process of clustering SCVs with similar activation patterns into the same cluster, resulting aggregated covariance matrix $\bar{C}$ is displayed in (**a**). The identified subgroups and the corresponding t-maps from the Gershgorin disc-based method and the compared method in [34] are shown in (**b**,**c**) separately. Subgroups 1 and 2 are denoted by yellow and magenta squares separately. Subgroups from both methods show significant activity differences in meaningful brain areas such as the dorsolateral prefrontal cortex (dlPFC), anterior cingulate cortex (ACC), etc. The Gershgorin disc-based method provides more meaningful brain areas that show significant group differences and the subgroups that form better block structures in the aggregated covariance matrix as is shown in ${\bar{C}}_{n}\left(g\right)$ than the one formed by the compared method in ${\bar{C}}_{n}\left(m\right)$.

The identified subgroups, the brain areas that show significant group differences, and their corresponding statistical tests results are shown in Figure 4 and listed in Table 1, Table 2, Table 3 and Table 4. As mentioned in Section 2.3, after the clustering of SCVs, the aggregated SCV covariance matrix $\bar{C}$ is achieved by taking the average of the SCV covariance matrices within the same cluster. Two subgroup detection methods, the Gershgorin disc-based method [35] and the method in [34], are implemented and compared. In [35], eigenanalysis based on Gershgorin disc was proposed to determine the number of subgroups and the subjects belonging to each subgroup. For a given covariance matrix ${\hat{C}}_{n}$, the sum of the absolute values of the non-diagonal entries in the ith row is represented as $R_{i}=\sum_{j\neq i}|\rho_{n}^{\left[i,j\right]}|$. A Gershgorin disc is defined as a closed disc centered at $\rho_{n}^{\left[i,i\right]}$ with radius $R_{i}$, $\left\{z\in R:|z-\rho_{n}^{\left[i,i\right]}|\leq R_{i}\right\}$, where $\rho_{n}^{\left[i,i\right]}$ is the entry on the ith row and ith column of ${\hat{C}}_{n}$. Let $R_{\min}$ be the radius of the smallest Gershgorin disc. The number of subgroups is identified as the number of eigenvalues of ${\hat{C}}_{n}$ that is located outside the smallest Gershgorin disc. For a normalized ${\hat{C}}_{n}$ with unit variance, its diagonal entries are 1, i.e., $\rho_{n}^{\left[i,i\right]}=1$. Therefore, eigenvalues that are located outside the smallest Gershgorin disc should be greater than $R_{\min}+1.$ The subjects belonging to each subgroup are identified by applying *k*-mean clustering on the corresponding eigenvectors. The method presented in [35] identifies the subgroups by maximizing the modularity of the given covariance matrix ${\hat{C}}_{n}$. More details about applying the Gershgorin disc-based method to an aggregated sample covariance matrix $\bar{C}$ can be found in Supplementary Figure S1.

As it is shown in Figure 4, Gershgorin disc-based approach reveals a better subgroup structure than the method in [34] as the block structure is more obvious in the Gershgorin disc-based result. This can be explained by the nature of the Gershgorin disc-based method that takes the eigenspectrum of $\bar{C}$ into consideration. The statistical tests on neuroimaging and cognitive variables also show more significant group differences with the Gershgorin disc-based method results. Three neuronetwork components that show significant group differences are found by the Gershgorin disc-based method versus one component by the method in [34]. We investigate the cognitive scores by applying MANOVA to each set of tests. The MANOVA results for the Gershgorin disc-based method are *F*-score = 4.95 ($p=1.5\times 10^{-4}$) for BACS, *F*-score = 5.3 ($p=7.21\times 10^{-6}$) for SFS, and *F*-score = 7.57 ($p=6.01\times 10^{-25}$) for PANSS separately. Two-sample *t*-test is implemented on each subtest to assess differences between the subgroups. The identified subgroups from the Gershgorin disc-based method have shown significant group differences in the majority of subtests. The two-sample *t*-test results of each subtest are reported in Table 2, Table 3 and Table 4, respectively.

As shown in Figure 4b, the Gershgorin disc-based method detected three components that show group differences in the spatial activation patterns. The subgroup identified by the Gershgorin disc-based method has higher activation in the following brain areas relative to the other subjects: the dorsolateral prefrontal cortex (dlPFC), Broca’s area, primary somatosensory cortex, primary auditory, middle temporal gyrus, superior temporal gyrus, supramarginal gyrus, and anterior cingulate cortex (ACC). The corresponding description of these brain areas are defined using the Brodmann Area (BA) and Montreal Neurological Institute and Hospital (MNI) coordinate system [76,77] in Table 1. Similar activation areas that show group differences can be found in Figure 4c from the subgroups that are identified by the modularization method [34]. However, the results from the Gershgorin disc-based method reveal more functional network components that show group differences and have lower *p*-values, e.g., the primary auditory area.

As shown in Figure 4b, the identified subgroup (marked by a yellow square) shows significant group differences with the rest of the subjects (subgroup 2, marked by magenta square) in a variety of brain areas that have shown to be related to schizophrenia, psychotic bipolar disorder, and schizoaffective diseases. One of these brain areas is dlPFC, which is part of the central executive network that is involved in multiple cognitive functions including working memory [78,79], verbal fluency [80], etc. It is shown that patients with psychiatric disorders experience obvious deficits in their working memory and have a dysfunctional dlPFC area [81]. Studies in [47,82] show that performances on digit sequencing tests and verbal fluency tests are linked with working memory function. As shown in Table 2, the digit sequencing test and verbal fluency test from BACS show significant group differences between the identified groups. Subjects in the identified subgroup have a higher mean value (−1.01±1.29) on the BACS composite score than the rest of the subjects (−1.59±1.36).

Another meaningful brain area that shows significant group difference is ACC, which is part of a large-scale brain network, the salience network, which has contributions to complex functions such as communication, social behavior, etc. [83]. The connection between the abnormalities of the anterior cingulate cortex and multiple psychiatric disorders is studied in [43,44]. The statistical test results of SFS scores are also aligned with the previous research that the subjects in the identified subgroups have better social skills than the rest of the subjects. For example, the Prosocial Performance and Occupation/Employment scores show great group differences. Furthermore, the mean value of the SFS total score is 133.79±20.65 for the subjects within the identified subgroup, and 123.97±23.19 for the rest of the subjects.

The superior temporal gyrus is another identified important brain area where abnormalities have been found connected to language-related symptoms in schizophrenia patients [45]. The severity of dysconnectivity in supramarginal gyrus shows different levels of processing speed deficits in schizophrenia and psychotic bipolar disorder [84]. The symbol coding test from BACS assesses the attention and speed of information processing ability of the subject. The mean score of the symbol coding test of the identified subgroup is −1.01±1.06 and −1.28±1.15 for the rest of the subjects, which coincides with the aforementioned neuroimaging results.

The validation from cognitive test scores brings a complete picture of the identified subgroup. The subjects within the subgroup show higher activation in the brain areas such as dlPFC, ACC, and superior temporal gyrus, and these subjects experience fewer functional deficits in working memory, verbal fluency, and processing speed. The combination of the clear block structure revealed in $\bar{C}$, the meaningful functional brain areas, and the high significance level, i.e., small *p*-value, of each subtest give high confidence in the identified subgroup.

## 4. Discussion

Identifying subgroups through the activation patterns of the RSNs is an effective method to find subjects that show homogeneity in their neuropsychology profiles and therefore also homogeneity in their symptoms [34,35]. The accessible large-scale multi-subject neuroimaging data provide new opportunities for studying homogeneous subgroups of psychiatric disorders objectively. Along with subjects’ cognitive test scores, the identified subgroups are thoroughly verified by utilizing both neuroimaging and cognitive test data.

In this study, we demonstrate the effectiveness of applying the proposed pipeline, fSIG, to identify homogeneous subgroups from a large-scale fMRI dataset. By using a constrained version of ICA, the connection of brain activities across subjects is established through the constraint. We also illustrate the process of extracting stable RSN templates that can be used for c-ICA, which is a critical step for any c-ICA algorithms. From the brain activities estimated by c-ICA, a Gershgorin disc-based method is used to explore the homogeneous subgroup structures.

The identified subgroups from fSIG show significant group differences in multiple meaningful brain areas including the dorsolateral prefrontal cortex, Broca-Operc, primary somatosensory cortex, primary auditory, middle temporal gyrus, superior temporal gyrus, supramarginal gyrus, and anterior cingulate cortex. These brain areas have been reported in earlier research to be insightful biomarkers of various psychiatric disorders. Three sets of cognitive test scores are included in the study to verify the validation of the identified subgroups. The observed abnormality in the aforementioned brain areas is consistent with the functional deficits observed from the cognitive test scores. These observations also show that patients in the identified subgroup from the B-SNIP dataset experience less severe symptoms and brain function deficits compared with the rest of the patients.

We find it interesting that the identified subgroups from the Gershgorin disc-based method show significant group differences in both dlPFC and ACC areas. The abnormalities observed in dlPFC and ACC areas are consistent with the functional deficits observed in the cognitive test scores. Studies from [85,86] show that the functional networks to which dlPFC and ACC belong, the central executive network and salience network, play critical roles in connecting heterogeneous symptoms of psychiatric disorders with the pathophysiological mechanism. This confirms the significant group differences observed from the PANSS scores, which are shown in Table 4. The consistent results that are drawn from different types of data increase our confidence that the identified subgroups are meaningful. Compared with the method proposed in [34], the Gershgorin disc-based method shows better group structure in the covariance matrix of the SCVs and also lower *p*-values overall. The consistency that the results coming from two different methods coincide with each other increases the confidence in using the connection of brain activities to identify potential homogeneous subgroups.

We note that we only refer to two methods, the Gershgorin disc-based method and the method described in [34], for the second step of our subgroup identification problem. The main reason for this is that the identification of subgroups from fMRI or other large datasets is a crucial problem that has not yet been sufficiently addressed in the literature. To date, only two related works, namely the Gershgorin disc-based method and the method described in study [34], have utilized SCVs for subgroup identification in this research field. However, the previous work in [35] cannot be compared directly with the method in [34], as the Gershgorin disc-based method was applied to individual SCV covariance matrices. In this study, the proposed fSIG pipeline enables a direct comparison of these two methods. Besides the proposed pipeline and the improved computational efficiency, compared with previous work [34], our results demonstrate that the Gershgorin disc-based method identifies subgroups that exhibit more significant group differences in various cognitive scores than the other method.

Even though the current study shows promising results in identifying homogeneous subgroups, there are some areas worthy of further exploration. The RSNs references used for this study are generated from a single site fMRI data, COBRE, which can be improved by incorporating subjects from multiple sites. ICA-EBM is used for reference generation in this study and the impact of references generated by other ICA algorithms will be investigated in the future. The current c-EBM algorithm requires the specification of a constraint parameter, a variation of the method that can adaptively select a constraint value based on the statistical properties of the estimates, and the references might be desirable. The current results of the identified subgroups are based on the Gershgorin disc-based method and other types of identification approaches such as community detection are in the realm of research interests as well. We also noticed that the Gershgorin disc-based method has some limitations. For example, the number of subgroups identified by the Gershgorin disc-based method may be affected by the dimension of the SCV sample covariance matrix. When the dimension becomes large, the radius of the smallest Gershgorin disc increases, which can cause an underestimation of the number of subgroups. Supplementary Figure S2 displays the distribution of subjects with different diagnosis labels across the two subgroups that were identified. We note, however, that this distribution of subjects with different diagnosis labels does not provide a definitive characterization of the identified subgroups. Therefore, we have also used behavior variables or cognitive scores to validate the subgroups. In future studies, we aim to explore the identification of disease subtypes. Such subtype information would be valuable for identifying biomarkers and improving our understanding of the etiology of different diseases. Given the complexity of identifying subgroups in mental disorders, the continued development of effective subgroup identification methods from large-scale fMRI data is needed for future research.

## Figures and Tables

**Table 1:** Peak activations in the *t*-maps of the resting-state networks that show group differences.

Name	BA	MNI
dlPFC	9	−45, 33, 33
Broca-Operc	44	−60, 6, 18
Primary somatosensory cortex	1	42, −42, 63
Primary auditory	41	63, −27, 9
Middle temporal gyrus	21	−51, −51, 6
Superior temporal gyrus	21	53, −40, 8
Supramarginal gyrus	40	48, −38, 57
ACC	24	0, 30, 18

**Table 2:** Two-sample *t*-test of each test included in BACS (lower *p*-value is marked in bold).

BACS	*p*-Value (Method in [34])	*p*-Value (fSIG)
List Learning	$6.23\times 10^{-3}$	$1.61\times 10^{-3}$
Digit Sequencing	$2.43\times 10^{-6}$	$5.98\times 10^{-6}$
Token Motor	$4.19\times 10^{-2}$	$3.43\times 10^{-3}$
Verbal Fluency	$8.87\times 10^{-4}$	$1.60\times 10^{-2}$
Symbol Coding	$1.09\times 10^{-2}$	$9.67\times 10^{-3}$
Tower of London	$2.00\times 10^{-4}$	$1.64\times 10^{-4}$

**Table 3:** Two-sample *t*-test of each field included in SFS (lower *p*-value is marked in bold and non-significant values are marked with ${}^{*}$).

SFS	*p*-Value (Method in [34])	*p*-Value (fSIG)
Social Engagement	8.48 × 10${}^{-1}$ ${}^{*}$	**9.46**×**10**${}^{-2}$${}^{*}$
Interpersonal Communication	8.00 × 10${}^{-5}$	**5.71**×**10**${}^{-5}$
Independence /Competence	1.41 × 10${}^{-1}$ ${}^{*}$	**2.57**×**10**${}^{-3}$
Recreation Performance	5.51 × 10${}^{-2}$ ${}^{*}$	**4.41**×**10**${}^{-5}$
Independence/Performance	1.94 × 10${}^{-2}$	**4.88**×**10**${}^{-5}$
Prosocial Performance	3.17 × 10${}^{-3}$	**7.57**×**10**${}^{-6}$
Occupation/Employment	2.49 × 10${}^{-1}$ ${}^{*}$	**1.71**×**10**${}^{-3}$

**Table 4:** Two-sample *t*-test of the positive, negative, and general symptoms in PANSS (lower *p*-Value is marked in bold and non-significant values are marked with ${}^{*}$).

PANSS	*p*-Value (Method in [34])	*p*-Value (fSIG)
Positive Total	3.81 × 10${}^{-3}$	**9.56**×**10**${}^{-14}$
Negative Total	1.79 × 10${}^{-8}$	**6.83**×**10**${}^{-12}$
General Total	8.45 × 10${}^{-1}$ ${}^{*}$	**6.86**×**10**${}^{-4}$

## Data Availability

The data from the Center of Biomedical Research Excellence (COBRE) is accessible from https://coins.trendscenter.org/, (accessed on 10 January 2023). The data from Bipolar-Schizophrenia Network on Intermediate Phenotypes (B-SNIP) is accessible from https://nda.nih.gov/edit_collection.html?id=2274, (accessed on 10 January 2023).

## Supplementary Materials

The following supporting information can be downloaded at: https://www.mdpi.com/article/10.3390/s23063264/s1.

## Abbreviations

The following abbreviations are used in this manuscript:

ICA	Independent component analysis
GICA	Group independent component analysis
c-ICA	Constrained independent component analysis
EBM	Entropy bound minimization
IVA	Independent vector analysis
SCV	Source component vector
RSN	Resting-state network
GDM	Global difference map
Cross-ISI	Cross inter-symbol interference
