# Supplementary material for: Identification of Homogeneous Subgroups from Resting-State fMRI Data
Source: Sensors (Basel). 2023 Mar 20;23(6):3264. doi: 10.3390/s23063264 (PMC10051904; doi:10.3390/s23063264)
Supplement: Supplementary file 1 [file sensors-23-03264-s001.zip › sensors-2186514-supplementary.pdf]

Identification of Homogeneous Subgroups from Resting-State fMRI Data (Supplemental Materials)

Hanlu Yang [1,*], Trung Vu [1], Qunfang Long [1], and Vince Calhoun [2], Tülay Adali [1,*]

[1] Department of Computer Science and Electrical Engineering, University of Maryland Baltimore County

Baltimore, MD 21250, USA;

[2] Tri-Institutional Center for Translational Research in Neuroimaging and Data Science (TReNDS)

Georgia State University, Georgia Institute of Technology, and Emory University

Atlanta, GA 30303, USA

* Correspondence: hyang3@umbc.edu (H.Y.); adali@umbc.edu (T.A.)

In the supplemental materials,

- we provide the details of the application of the Gershgorin disc-based method to the aggregated sample covariance matrix $\bar{\mathbf{C}}$ presented in the paper, including eigenvalues, the radius of the smallest Gershgorin disc and the corresponding eigenvectors in Figure S1.
- we include the distribution of subjects with diagnosis labels in each of the identified subgroups in Figure S2.
- we present the subgroup identification results from a number of other clusters as examples in Figure S3-S17, and the verification results based on the behavior data in Table S1- S9.

The results show that the Gershgorin disc-based method performs better than the method presented in [1] in identifying subgroups with a stronger group structure in the rearranged aggregated sample covariance matrix $\bar{\mathbf{C}}$ and yields more significant group differences in various cognitive test scores. To validate the identified subgroups from different perspectives, three sets of cognitive test scores (i.e., Social Functioning Scale (SFS) [2], Brief Assessment of Cognition in Schizophrenia (BACS) [3], and Positive and Negative Syndrome Scale (PANSS) [4]) are statistically analyzed. The results from fSIG demonstrate significant group differences in most cognitive test scores across the subgroups identified using neuroimaging data.

The application of the Gershgorin disc-based method

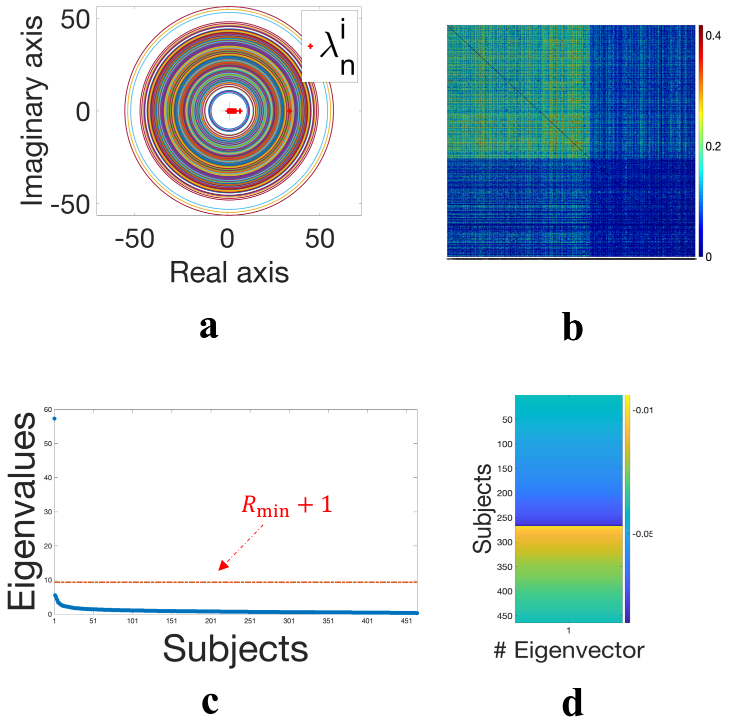

Figure S1: Additional information about the application of the Gershgorin disc-based method to an aggregated sample covariance matrix $\bar{\mathbf{C}}$. Panel (a) illustrates the Gershgorin discs for a given $\bar{\mathbf{C}}$ and its corresponding eigenvalue locations, where the red dots represent the actual eigenvalues. Panel (b) displays the visualization for a given matrix $\bar{\mathbf{C}}$. Panel (c) shows the distribution of eigenvalues for the same $\bar{\mathbf{C}}$, and the red line is set to $R_{\min} + 1$, where $R_{\min}$ is the radius of the smallest Gershgorin disc. In panel (d), we present the eigenvector that corresponds to the eigenvalue located outside the smallest Gershgorin disc. After permuting, a clear group structure emerges, with the subjects in the first subgroup exhibiting overall higher values of the corresponding eigenvector than those in the second subgroup.

The distribution of subjects with diagnosis labels

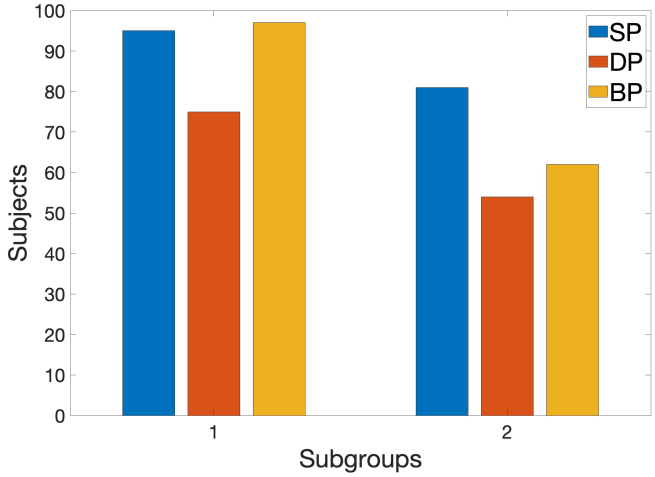

Figure S2: Distribution of subjects with different diagnosis labels in the two identified subgroups. The abbreviations used are: SP for Schizophrenia patients, DP for schizoaffective disorder patients, and BP for Bipolar Disorder. However, this distribution of subjects with different diagnosis labels is not definitive on the identified subgroups, and hence we have used the behavior variables or cognitive scores for subgroups' validation.

Additional Subgroup Identification Results

Cluster # 1

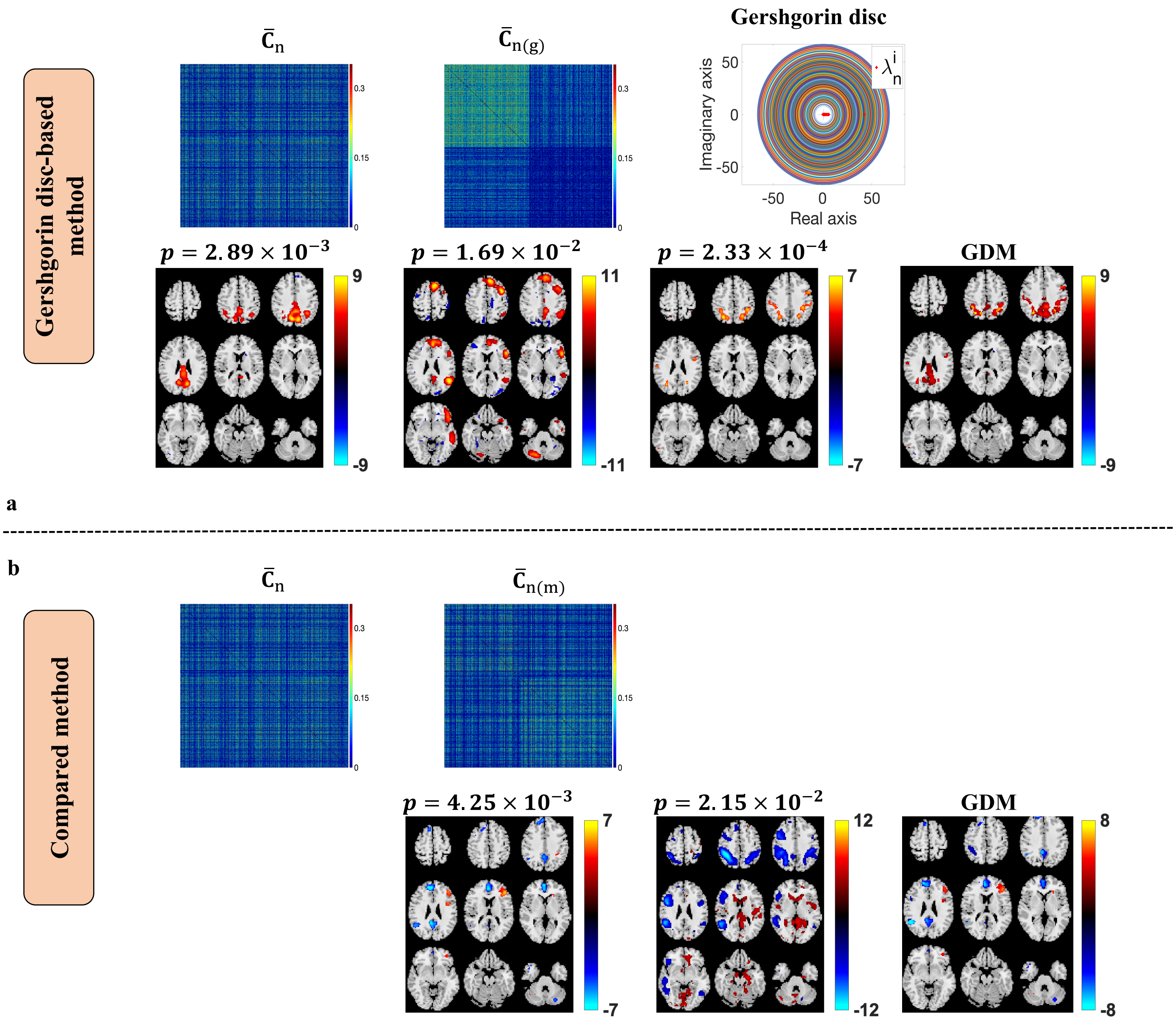

Figure S3: The identified subgroups and the corresponding t-maps from the Gershgorin disc-based method and the compared method in [1] are shown in (a) and (b) separately. The Gershgorin disc-based method provides more meaningful brain areas that show significant group differences and the subgroups that form better block structures in the aggregated covariance matrix as is shown in $\bar{\mathbf{C}}_n(g)$ than the one formed by the compared method in $\bar{\mathbf{C}}_n(m)$.

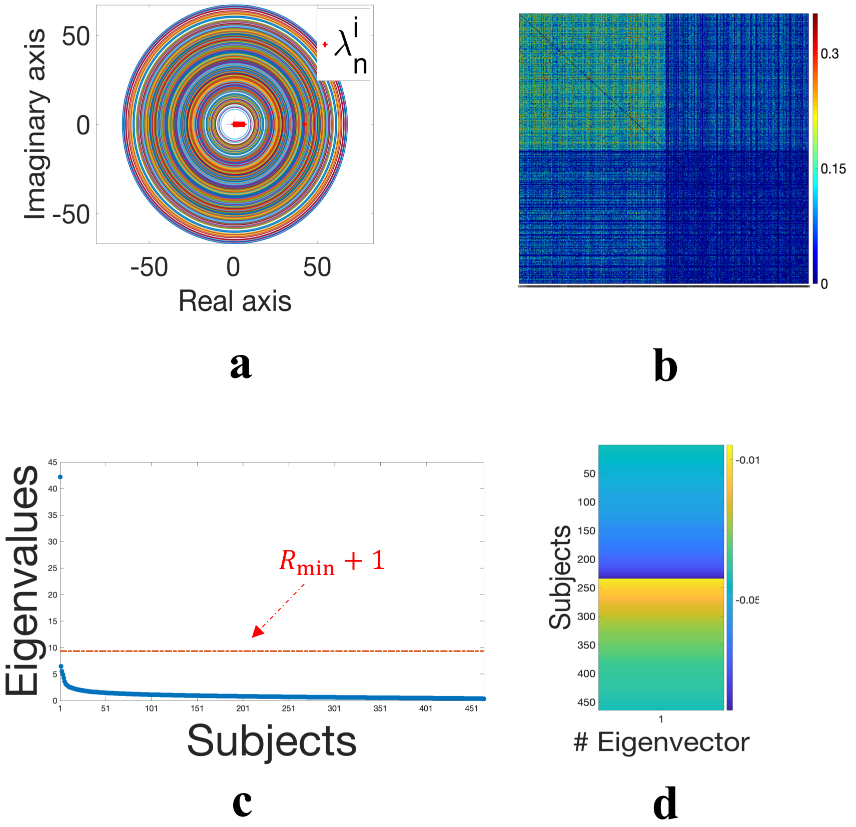

Figure S4: Additional information about the application of the Gershgorin disc-based method to an aggregated sample covariance matrix $\bar{\mathbf{C}}$. Panel (a) illustrates the Gershgorin discs for a given $\bar{\mathbf{C}}$ and its corresponding eigenvalue locations, where the red dots represent the actual eigenvalues. Panel (b) displays the visualization for a given matrix $\bar{\mathbf{C}}$. Panel (c) shows the distribution of eigenvalues for the same $\bar{\mathbf{C}}$, and the red line is set to $R_{\min} + 1$, where $R_{\min}$ is the radius of the smallest Gershgorin disc. In panel (d), we present the eigenvector that corresponds to the eigenvalue located outside the smallest Gershgorin disc. After permuting, a clear group structure emerges, with the subjects in the first subgroup exhibiting overall higher values of the corresponding eigenvector than those in the second subgroup.

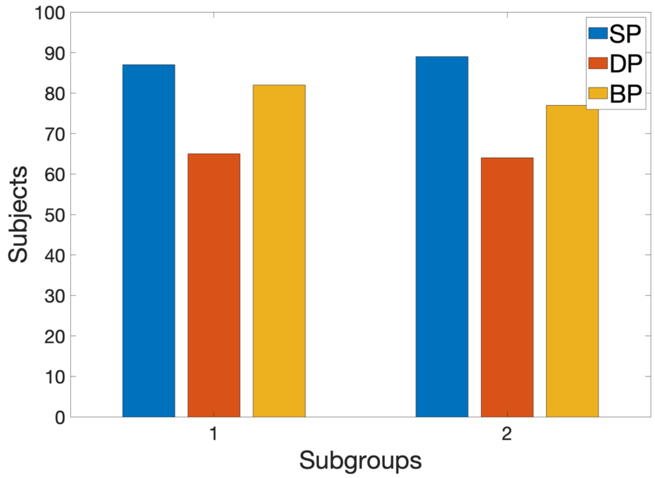

Figure S5: Distribution of subjects with different diagnosis labels in the two identified subgroups. The abbreviations used are: SP for Schizophrenia patients, DP for schizoaffective disorder patients, and BP for Bipolar Disorder.

Cluster # 2

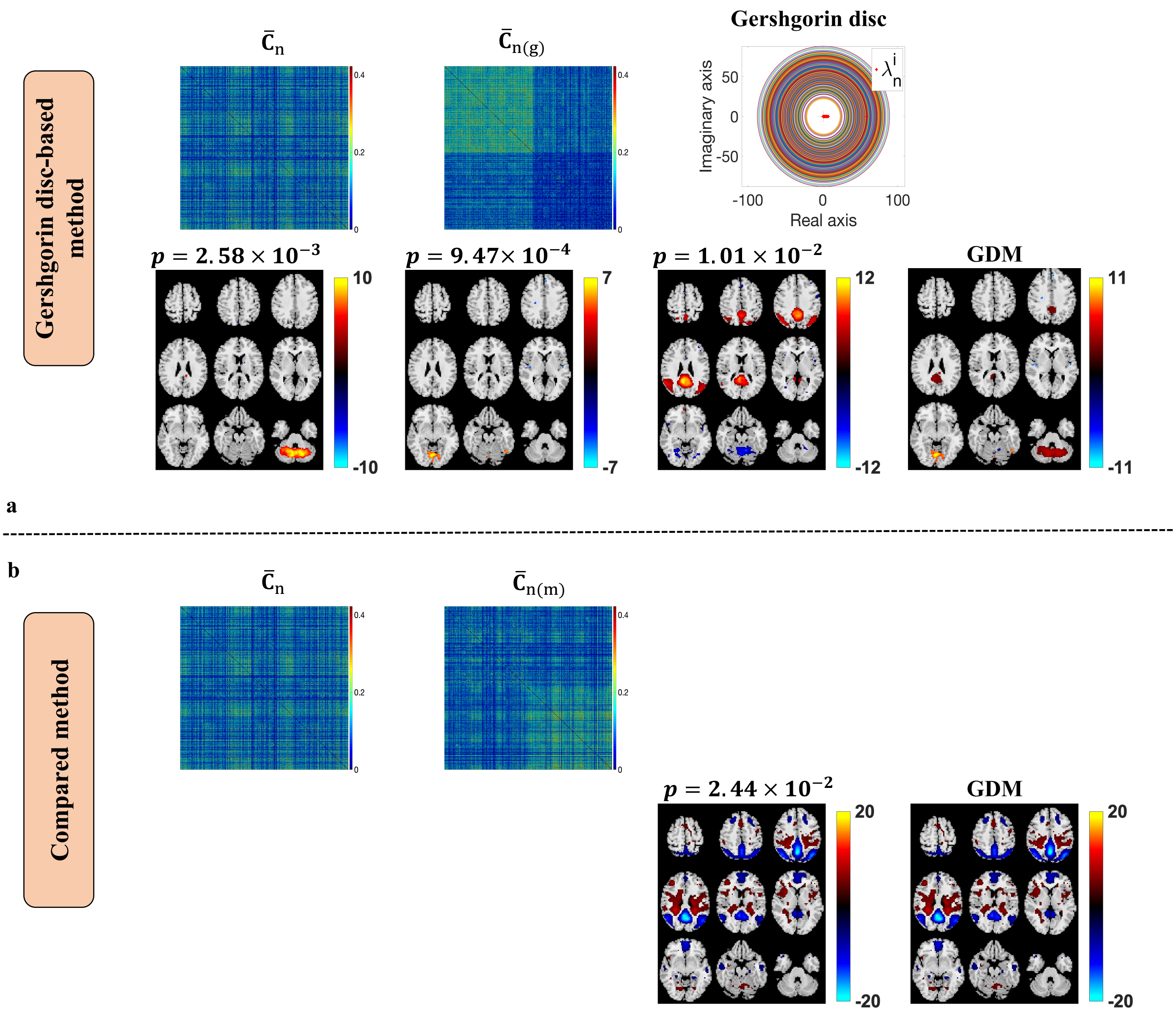

Figure S6: The identified subgroups and the corresponding t-maps from the Gershgorin disc-based method and the compared method in [1] are shown in (a) and (b) separately. The Gershgorin disc-based method provides more meaningful brain areas that show significant group differences and the subgroups that form better block structures in the aggregated covariance matrix as is shown in $\bar{\mathbf{C}}_n(g)$ than the one formed by the compared method in $\bar{\mathbf{C}}_n(m)$.

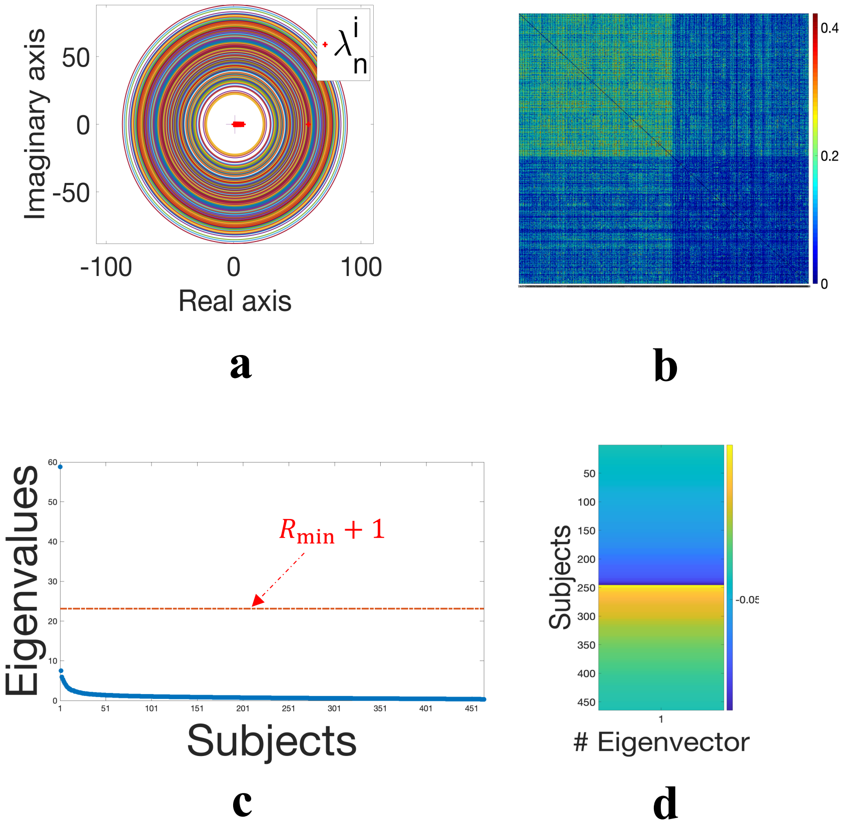

Figure S7: Additional information about the application of the Gershgorin disc-based method to an aggregated sample covariance matrix $\bar{\mathbf{C}}$. Panel (a) illustrates the Gershgorin discs for a given $\bar{\mathbf{C}}$ and its corresponding eigenvalue locations, where the red dots represent the actual eigenvalues. Panel (b) displays the visualization for a given matrix $\bar{\mathbf{C}}$. Panel (c) shows the distribution of eigenvalues for the same $\bar{\mathbf{C}}$, and the red line is set to $R_{\min} + 1$, where $R_{\min}$ is the radius of the smallest Gershgorin disc. In panel (d), we present the eigenvector that corresponds to the eigenvalue located outside the smallest Gershgorin disc. After permuting, a clear group structure emerges, with the subjects in the first subgroup exhibiting overall higher values of the corresponding eigenvector than those in the second subgroup.

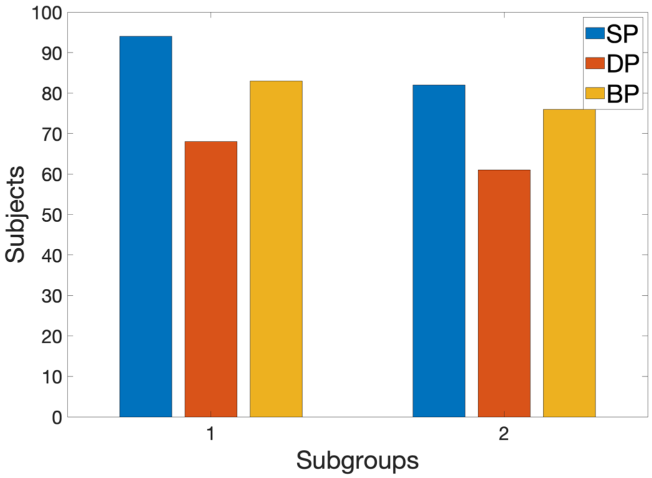

Figure S8: Distribution of subjects with different diagnosis labels in the two identified subgroups. The abbreviations used are: SP for Schizophrenia patients, DP for schizoaffective disorder patients, and BP for Bipolar Disorder.

Cluster # 3

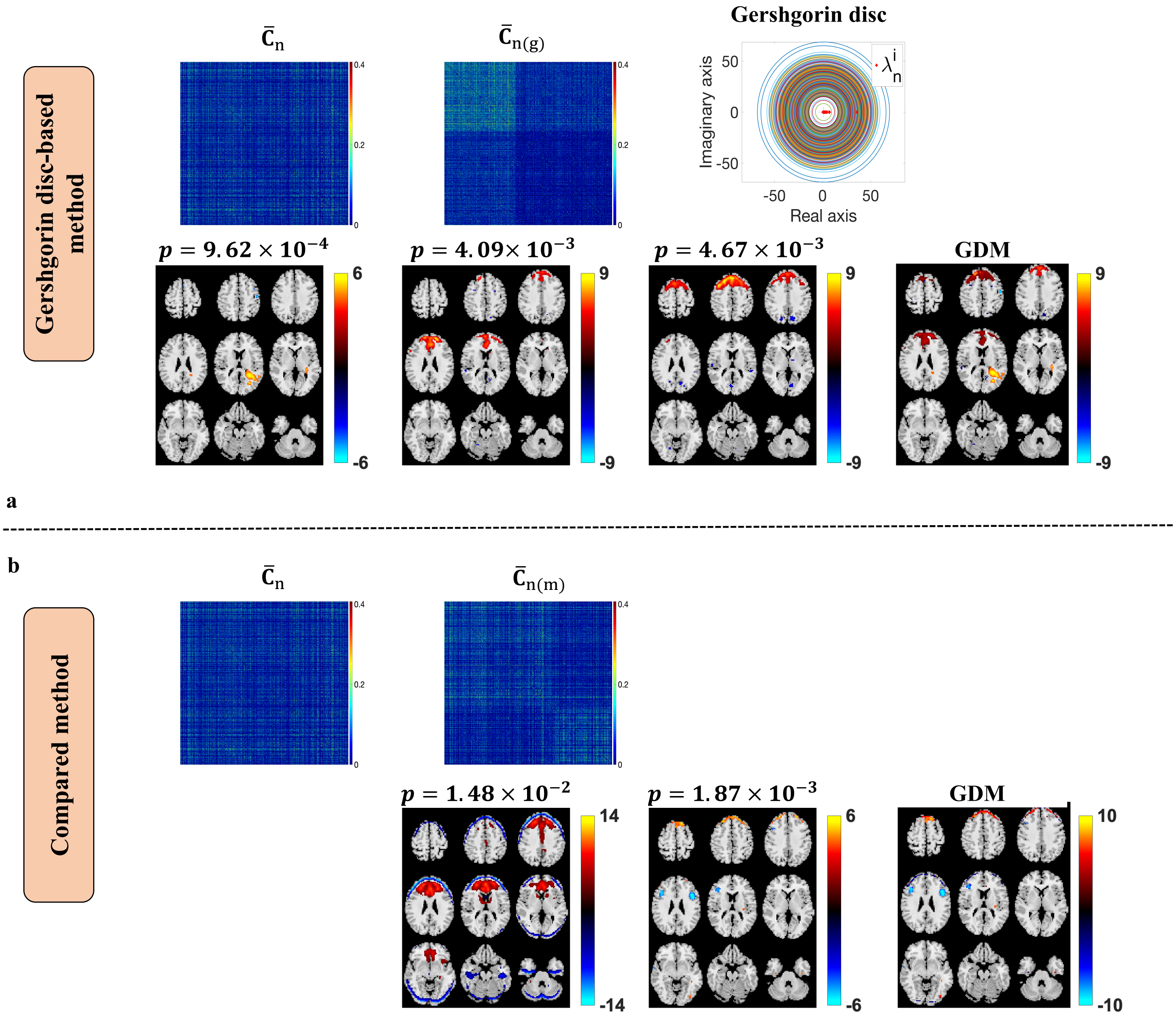

Figure S9: The identified subgroups and the corresponding t-maps from the Gershgorin disc-based method and the compared method in [1] are shown in (a) and (b) separately. The Gershgorin disc-based method provides more meaningful brain areas that show significant group differences and the subgroups that form better block structures in the aggregated covariance matrix as is shown in $\bar{\mathbf{C}}_n(g)$ than the one formed by the compared method in $\bar{\mathbf{C}}_n(m)$.

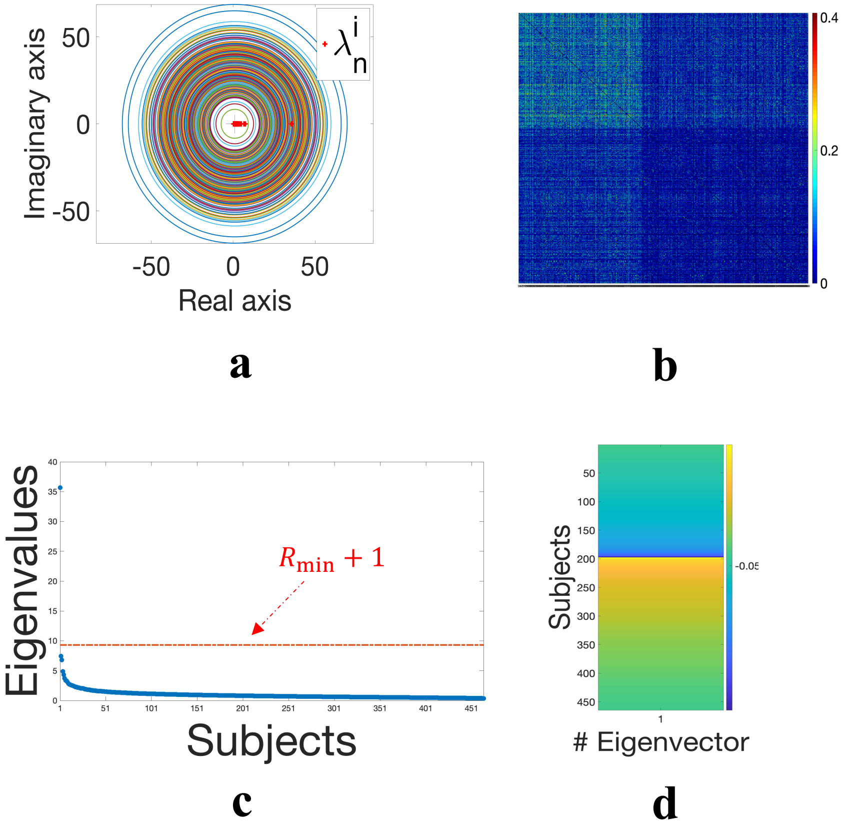

Figure S10: Additional information about the application of the Gershgorin disc-based method to an aggregated sample covariance matrix $\bar{\mathbf{C}}$. Panel (a) illustrates the Gershgorin discs for a given $\bar{\mathbf{C}}$ and its corresponding eigenvalue locations, where the red dots represent the actual eigenvalues. Panel (b) displays the visualization for a given matrix $\bar{\mathbf{C}}$. Panel (c) shows the distribution of eigenvalues for the same $\bar{\mathbf{C}}$, and the red line is set to $R_{\min} + 1$, where $R_{\min}$ is the radius of the smallest Gershgorin disc. In panel (d), we present the eigenvector that corresponds to the eigenvalue located outside the smallest Gershgorin disc. After permuting, a clear group structure emerges, with the subjects in the first subgroup exhibiting overall higher values of the corresponding eigenvector than those in the second subgroup.

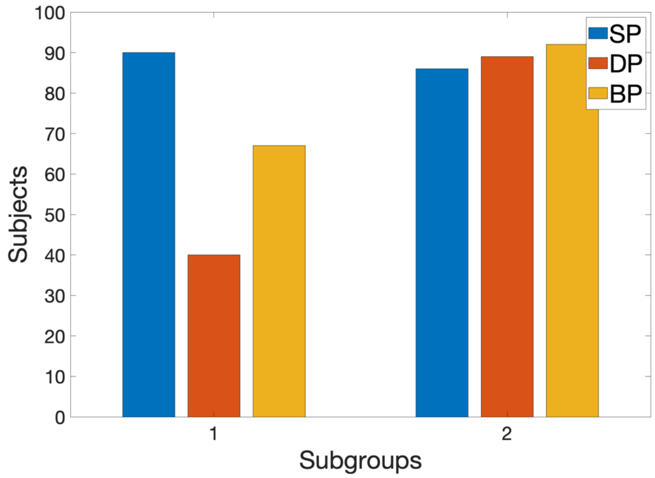

Figure S11: Distribution of subjects with different diagnosis labels in the two identified subgroups. The abbreviations used are: SP for Schizophrenia patients, DP for schizoaffective disorder patients, and BP for Bipolar Disorder.

Cluster # 4

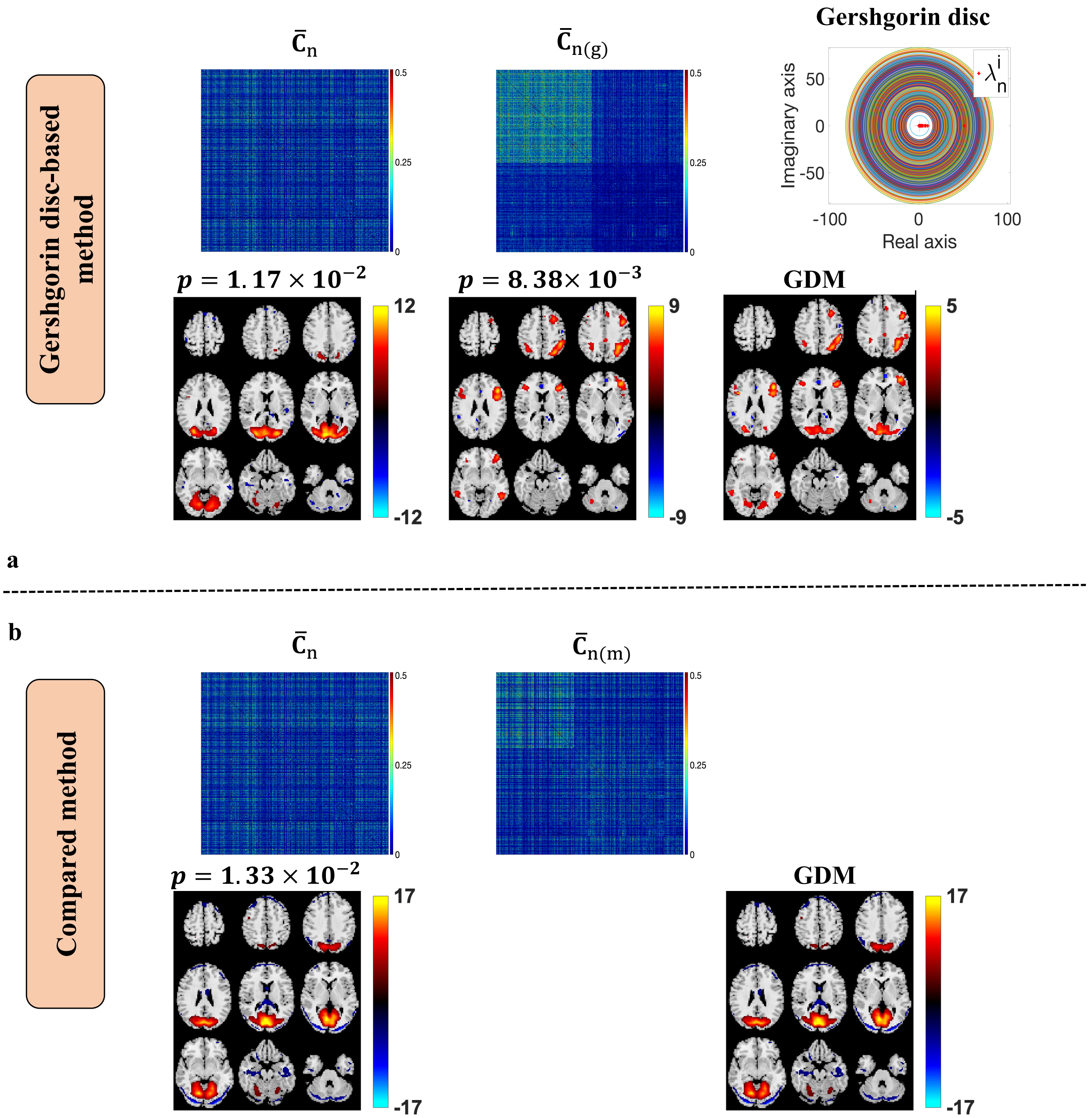

Figure S12: The identified subgroups and the corresponding t-maps from the Gershgorin disc-based method and the compared method in [1] are shown in (a) and (b) separately. The Gershgorin disc-based method provides more meaningful brain areas that show significant group differences and the subgroups that form better block structures in the aggregated covariance matrix as is shown in $\bar{\mathbf{C}}_n(g)$ than the one formed by the compared method in $\bar{\mathbf{C}}_n(m)$.

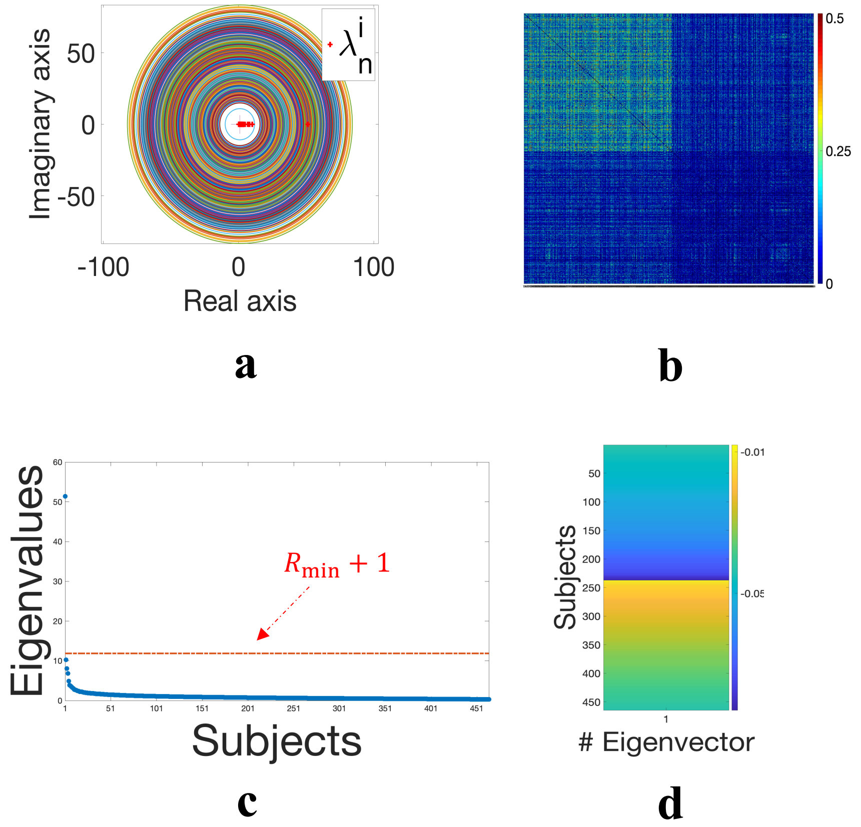

Figure S13: Additional information about the application of the Gershgorin disc-based method to an aggregated sample covariance matrix $\bar{\mathbf{C}}$. Panel (a) illustrates the Gershgorin discs for a given $\bar{\mathbf{C}}$ and its corresponding eigenvalue locations, where the red dots represent the actual eigenvalues. Panel (b) displays the visualization for a given matrix $\bar{\mathbf{C}}$. Panel (c) shows the distribution of eigenvalues for the same $\bar{\mathbf{C}}$, and the red line is set to $R_{\min} + 1$, where $R_{\min}$ is the radius of the smallest Gershgorin disc. In panel (d), we present the eigenvector that corresponds to the eigenvalue located outside the smallest Gershgorin disc. After permuting, a clear group structure emerges, with the subjects in the first subgroup exhibiting overall higher values of the corresponding eigenvector than those in the second subgroup.

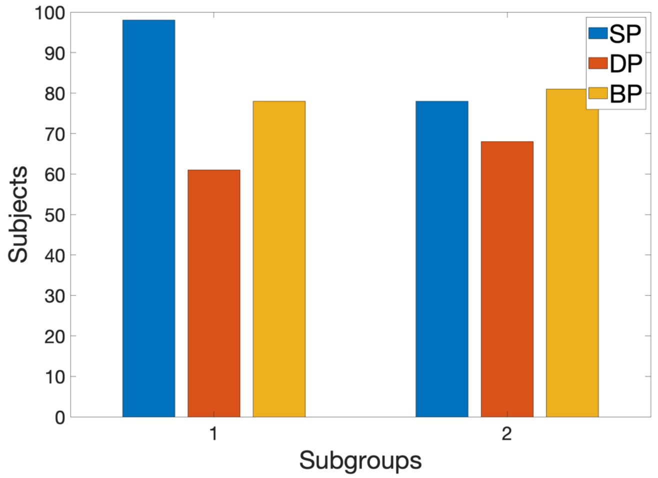

Figure S14: Distribution of subjects with different diagnosis labels in the two identified subgroups. The abbreviations used are: SP for Schizophrenia patients, DP for schizoaffective disorder patients, and BP for Bipolar Disorder.

Cluster # 5

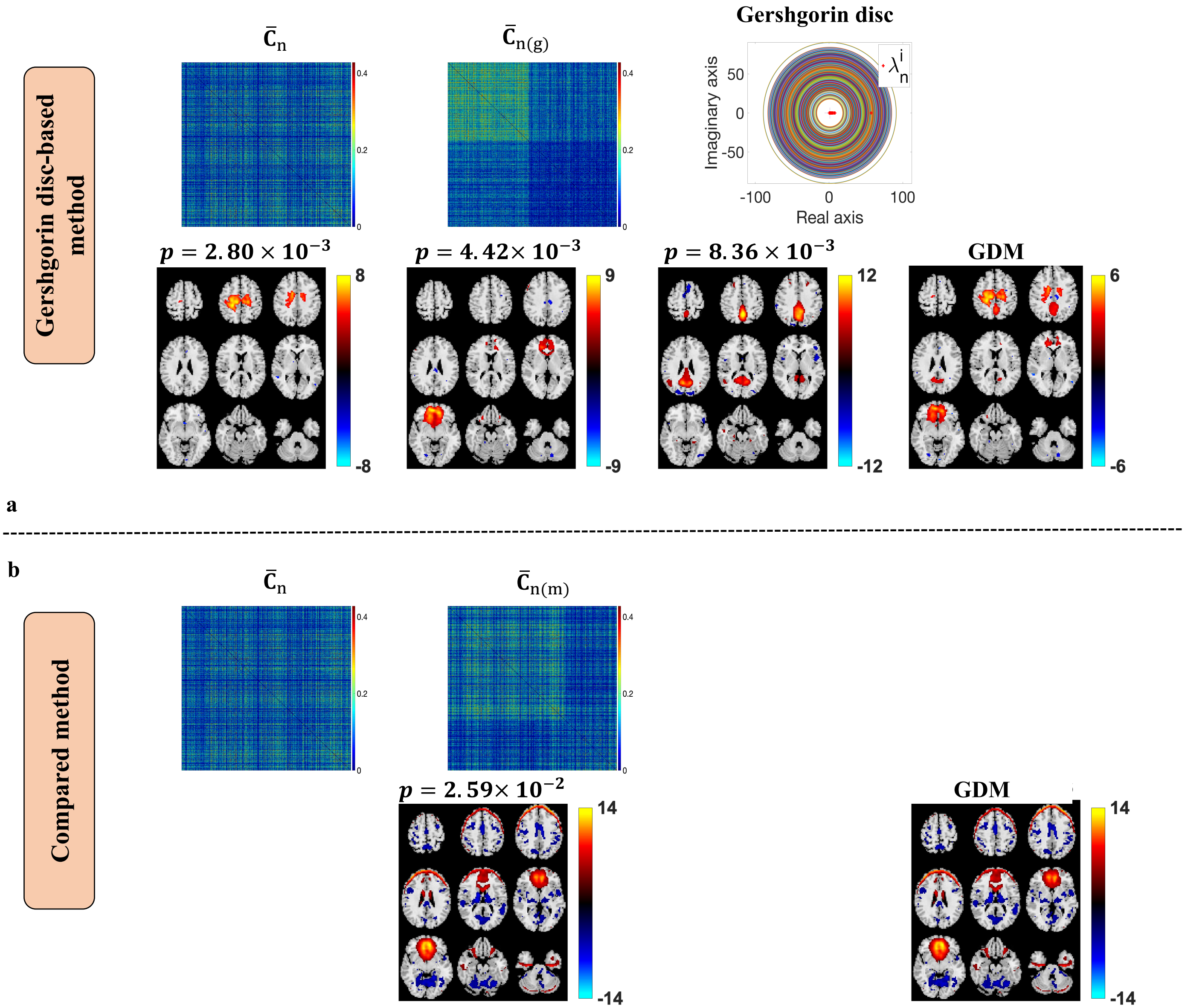

Figure S15: The identified subgroups and the corresponding t-maps from the Gershgorin disc-based method and the compared method in [1] are shown in (a) and (b) separately. The Gershgorin disc-based method provides more meaningful brain areas that show significant group differences and the subgroups that form better block structures in the aggregated covariance matrix as is shown in $\bar{\mathbf{C}}_n(g)$ than the one formed by the compared method in $\bar{\mathbf{C}}_n(m)$.

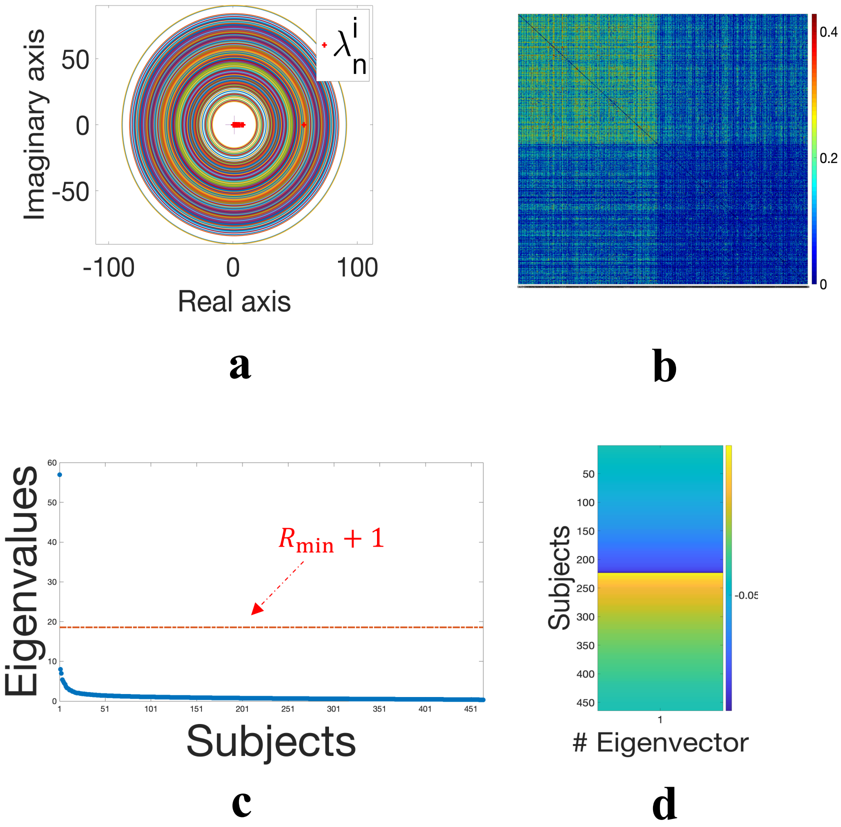

Figure S16: Additional information about the application of the Gershgorin disc-based method to an aggregated sample covariance matrix $\bar{\mathbf{C}}$. Panel (a) illustrates the Gershgorin discs for a given $\bar{\mathbf{C}}$ and its corresponding eigenvalue locations, where the red dots represent the actual eigenvalues. Panel (b) displays the visualization for a given matrix $\bar{\mathbf{C}}$. Panel (c) shows the distribution of eigenvalues for the same $\bar{\mathbf{C}}$, and the red line is set to $R_{\min} + 1$, where $R_{\min}$ is the radius of the smallest Gershgorin disc. In panel (d), we present the eigenvector that corresponds to the eigenvalue located outside the smallest Gershgorin disc. After permuting, a clear group structure emerges, with the subjects in the first subgroup exhibiting overall higher values of the corresponding eigenvector than those in the second subgroup.

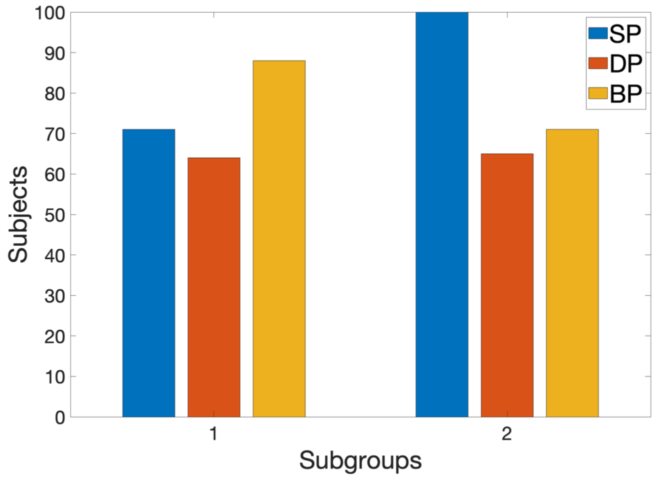

Figure S17: Distribution of subjects with different diagnosis labels in the two identified subgroups. The abbreviations used are: SP for Schizophrenia patients, DP for schizoaffective disorder patients, and BP for Bipolar Disorder.

Cluster # 1

Table S1: Two-sample t-test of each test included in BACS

**BACS**	p-**value** (Method in [1])	p-**value** (fSIG)
List Learning	$\mathbf{8.87 \times 10^{-4}}$	2.10×10^{-3}
Digit Sequencing	$\mathbf{2.82 \times 10^{-7}}$	4.35×10^{-7}
Token Motor	2.66×10^{-2}	$\mathbf{1.39 \times 10^{-2}}$
Verbal Fluency	2.69×10^{-4}	$\mathbf{6.36 \times 10^{-5}}$
Symbol Coding	1.70×10^{-3}	$\mathbf{1.40 \times 10^{-3}}$
Tower of London	8.79×10^{-4}	$\mathbf{1.75 \times 10^{-4}}$

Table S2: Two-sample t-test of each field included in SFS (non-significant values are marked with *).

**SFS**	p-**value** (Method in [1])	p-**value** (fSIG)
Social Engagement	$9.10 \times 10^{-1*}$	$\mathbf{2.54 \times 10^{-1}}^*$
Interpersonal Communication	1.80×10^{-4}	$\mathbf{6.09 \times 10^{-5}}$
Independence /Competence	$\mathbf{3.67 \times 10^{-1}}^*$	$6.63 \times 10^{-1*}$
Recreation Performance	$6.71 \times 10^{-2*}$	$\mathbf{2.49 \times 10^{-2}}$
Independence/Performance	$\mathbf{9.21 \times 10^{-2}}^*$	$2.33 \times 10^{-1*}$
Prosocial Performance	1.90×10^{-3}	$\mathbf{4.85 \times 10^{-4}}$
Occupation/Employment	$1.93 \times 10^{-1*}$	$\mathbf{4.26 \times 10^{-2}}$

Table S3: Two-sample t-test of the positive, negative, and general symptoms in PANSS (non-significant values are marked with *).

**PANSS**	p-**value** (Method in [1])	p-**value** (fSIG)
Positive Total	4.33×10^{-9}	$\mathbf{1.62 \times 10^{-12}}$
Negative Total	1.20×10^{-6}	$\mathbf{1.11 \times 10^{-7}}$
General Total	3.79×10^{-8}	$\mathbf{1.71 \times 10^{-9}}$

Cluster # 2

Table S4: Two-sample t-test of each test included in BACS

**BACS**	p-**value** (Method in [1])	p-**value** (fSIG)
List Learning	1.10×10^{-3}	$\mathbf{5.89 \times 10^{-4}}$
Digit Sequencing	$\mathbf{2.29 \times 10^{-7}}$	7.27×10^{-7}
Token Motor	4.51×10^{-2}	$\mathbf{1.31 \times 10^{-2}}$
Verbal Fluency	$\mathbf{1.68 \times 10^{-4}}$	2.20×10^{-3}
Symbol Coding	$\mathbf{2.54 \times 10^{-3}}$	3.65×10^{-3}
Tower of London	5.89×10^{-4}	$\mathbf{3.88 \times 10^{-4}}$

Table S5: Two-sample t-test of each field included in SFS (non-significant values are marked with *).

**SFS**	p-**value** (Method in [1])	p-**value** (fSIG)
Social Engagement	$\mathbf{3.07 \times 10^{-1}}^*$	$3.59 \times 10^{-1}*$
Interpersonal Communication	$\mathbf{7.97 \times 10^{-5}}$	9.20×10^{-5}
Independence /Competence	$5.60 \times 10^{-1}*$	$\mathbf{4.12 \times 10^{-1}}^*$
Recreation Performance	$3.14 \times 10^{-2}*$	$\mathbf{1.59 \times 10^{-2}}$
Independence/Performance	$\mathbf{1.57 \times 10^{-1}}$	$2.02 \times 10^{-1}*$
Prosocial Performance	4.83×10^{-4}	$\mathbf{9.12 \times 10^{-5}}$
Occupation/Employment	4.81×10^{-2}	$\mathbf{1.36 \times 10^{-2}}$

Table S6: Two-sample t-test of the positive, negative, and general symptoms in PANSS (non-significant values are marked with *).

**PANSS**	p-**value** (Method in [1])	p-**value** (fSIG)
Positive Total	9.44×10^{-13}	$\mathbf{7.13 \times 10^{-15}}$
Negative Total	6.97×10^{-8}	$\mathbf{1.59 \times 10^{-8}}$
General Total	$\mathbf{4.06 \times 10^{-10}}$	2.33×10^{-9}

Cluster # 4

Table S7: Two-sample t-test of each test included in BACS

**BACS**	p**-value** (Method in [1])	p**-value** (fSIG)
List Learning	1.98×10^{-3}	$\mathbf{1.17 \times 10^{-3}}$
Digit Sequencing	$\mathbf{8.09 \times 10^{-8}}$	1.29×10^{-6}
Token Motor	3.72×10^{-2}	$\mathbf{2.80 \times 10^{-2}}$
Verbal Fluency	$\mathbf{2.48 \times 10^{-5}}$	8.23×10^{-4}
Symbol Coding	$\mathbf{1.32 \times 10^{-3}}$	4.67×10^{-3}
Tower of London	2.72×10^{-3}	$\mathbf{1.41 \times 10^{-3}}$

Table S8: Two-sample t-test of each field included in SFS (non-significant values are marked with *).

**SFS**	p**-value** (Method in [1])	p**-value** (fSIG)
Social Engagement	$5.60 \times 10^{-1*}$	$\mathbf{3.26 \times 10^{-1}}^*$
Interpersonal Communication	$\mathbf{2.51 \times 10^{-5}}$	4.62×10^{-5}
Independence /Competence	$9.00 \times 10^{-1*}$	$\mathbf{6.46 \times 10^{-1}}^*$
Recreation Performance	$8.69 \times 10^{-2*}$	$\mathbf{2.50 \times 10^{-2}}$
Independence/Performance	$\mathbf{1.75 \times 10^{-1}}^*$	$2.59 \times 10^{1*}$
Prosocial Performance	7.00×10^{-3}	$\mathbf{2.05 \times 10^{-4}}$
Occupation/Employment	$2.47 \times 10^{-1*}$	$\mathbf{5.53 \times 10^{-2}}^*$

Table S9: Two-sample t-test of the positive, negative, and general symptoms in PANSS (non-significant values are marked with *).

**PANSS**	p**-value** (Method in [1])	p**-value** (fSIG)
Positive Total	3.18×10^{-7}	$\mathbf{3.25 \times 10^{-13}}$
Negative Total	1.36×10^{-6}	$\mathbf{2.01 \times 10^{-8}}$
General Total	2.99×10^{-6}	$\mathbf{6.77 \times 10^{-9}}$

References

[1] Q. Long, S. Bhinge, V. D. Calhoun, and T. Adali, "Independent vector analysis for common subspace analysis: Application to multi-subject fMRI data yields meaningful subgroups of schizophrenia," *NeuroImage*, vol. 216, p. 116872, 2020.

[2] M. Birchwood, J. Smith, R. Cochrane, S. Wetton, and S. Copestake, "The social functioning scale the development and validation of a new scale of social adjustment for use in family intervention programmes with schizophrenic patients," *The British Journal of Psychiatry*, vol. 157, no. 6, pp. 853–859, 1990.

[3] R. S. Keefe, T. E. Goldberg, P. D. Harvey, J. M. Gold, M. P. Poe, and L. Coughenour, "The brief assessment of cognition in schizophrenia: reliability, sensitivity, and comparison with a standard neurocognitive battery," *Schizophrenia research*, vol. 68, no. 2-3, pp. 283–297, 2004.

[4] S. R. Kay, A. Fiszbein, and L. A. Opler, "The positive and negative syndrome scale (panss) for schizophrenia," *Schizophrenia bulletin*, vol. 13, no. 2, pp. 261–276, 1987.
